# The complete genome sequence of *Dickeya zeae* EC1 reveals substantial divergence from other *Dickeya* strains and species

**DOI:** 10.1186/s12864-015-1545-x

**Published:** 2015-08-04

**Authors:** Jianuan Zhou, Yingying Cheng, Mingfa Lv, Lisheng Liao, Yufan Chen, Yanfang Gu, Shiyin Liu, Zide Jiang, Yuanyan Xiong, Lianhui Zhang

**Affiliations:** Guangdong Province Key Laboratory of Microbial Signals and Disease Control, Department of Plant Pathology, South China Agricultural University, Guangzhou, 510642 People’s Republic of China; State Key laboratory for Biocontrol, Sun Yat-Sen University, Guangzhou, 510275 China; Institute of Molecular and Cell Biology, 61 Biopolis Drive, Singapore, 138673 Republic of Singapore

## Abstract

**Background:**

*Dickeya zeae* is a bacterial species that infects monocotyledons and dicotyledons. Two antibiotic-like phytotoxins named zeamine and zeamine II were reported to play an important role in rice seed germination, and two genes associated with zeamines production, i.e., *zmsA* and *zmsK*, have been thoroughly characterized. However, other virulence factors and its molecular mechanisms of host specificity and pathogenesis are hardly known.

**Results:**

The complete genome of *D. zeae* strain EC1 isolated from diseased rice plants was sequenced, annotated, and compared with the genomes of other *Dickeya* spp.. The pathogen contains a chromosome of 4,532,364 bp with 4,154 predicted protein-coding genes. Comparative genomics analysis indicates that *D. zeae* EC1 is most co-linear with *D. chrysanthemi* Ech1591, most conserved with *D. zeae* Ech586 and least similar to *D. paradisiaca* Ech703. Substantial genomic rearrangement was revealed by comparing EC1 with Ech586 and Ech703. Most virulence genes were well-conserved in *Dickeya* strains except Ech703. Significantly, the *zms* gene cluster involved in biosynthesis of zeamines, which were shown previously as key virulence determinants, is present in *D. zeae* strains isolated from rice, and some *D. solani* strains, but absent in other *Dickeya* species and the *D. zeae* strains isolated from other plants or sources. In addition, a DNA fragment containing 9 genes associated with fatty acid biosynthesis was found inserted in the *fli* gene cluster encoding flagellar biosynthesis of strain EC1 and other two rice isolates but not in other strains. This gene cluster shares a high protein similarity to the fatty acid genes from *Pantoea ananatis*.

**Conlusion:**

Our findings delineate the genetic background of *D. zeae* EC1, which infects both dicotyledons and monocotyledons, and suggest that *D. zeae* strains isolated from rice could be grouped into a distinct pathovar, i.e., *D. zeae* subsp. *oryzae*. In addition, the results of this study also unveiled that the *zms* gene cluster presented in the genomes of *D. zeae* rice isolates and *D. solani* strains, and the fatty acid genes inserted in the *fli* gene cluster of strain EC1 were likely derived from horizontal gene transfer during later stage of bacterial evolution.

**Electronic supplementary material:**

The online version of this article (doi:10.1186/s12864-015-1545-x) contains supplementary material, which is available to authorized users.

## Background

*Dickeya zeae* is the major pathogen responsible for the maize stalk rot and rice foot rot diseases, and has the ability to infect both monocotyledons and dicotyledons. Other members of *Dickeya* are *D. chrysanthemi*, *D. dianthicola*, *D. dieffenbachiae*, *D. paradisiaca*, *D. dadantii* and *D. solani* [1,2]. A recent *recA*-based molecular evolution and genetic diversity study showed that *D. zeae* is least related to other *Dickeya* species and even within *D. zeae*, there are many sequence variants (sequevars) among the isolates from different host plants [3], suggesting a long history of *D. zeae* adaptation and evolution in the processes of pathogen-host interactions.

The symptoms caused by *Dickeya* infection include soft rot as well as wilts resulting from vascular invasions. Extensive studies on the *Dickeya* pathogens which infect dicotyledon crops and ornamental plants led to identify a range of virulence factors including extracellular enzymes, phospholipase, iron metabolism, siderophores, indigoidine pigment, and type III secretion system, collectively contributing to the bacterial infections [4]. In contrast, genetic analysis and biochemical characterization of the pathogenic mechanisms of *D. zeae* were initiated only in recent years [5-8], following the outbreak of the rice root rot disease in China [9]. These studies led to identification of a new family of phytotoxins, i.e., zeamine and zeamine II [5,6], which appear to act as the key virulence determinants of the pathogen as mutation of the zeamine synthase genes *zmsA* and *zmsK* resulted in partial or almost complete loss of virulence on rice seeds germination [6,7]. The regulatory mechanisms that govern virulence gene expression are largely unknown, except that an acyl-homoserine lactone (AHL) quorum sensing (QS) system was shown to be involved in the regulation of certain virulence traits *in D. zeae*, including bacterial motility and biofilm development [8].

Up till now, four complete *Dickeya* bacterial genome sequences have become publically available, including *D. paradisiaca* Ech703 isolated from *Solanum tuberosum* [10] (sequence release date: June 26, 2009), *D. chrysanthemi* Ech1591 causing maize and *Zea mays* soft rot [10] (sequence release date: July 7, 2009), *D. zeae* Ech586 isolated from *Philodendron* [10] (sequence release date: December 11, 2009), and *D. dadantii* 3937 isolated from *Saintpaulia ionantha* [11] (sequence release date: September 16, 2010). Additionally, a range of partial genome sequences of *Dickeya* species have also been presented, including four *D. danthicola* strains (GBBC 2039, NCPPB 3534, NCPPB 453 and IPO 980), six *D. solani* strains (GBBC 2040, MK10, MK16, IPO 2222, Ds0432-1, and 3337) [12-14], three *D. chrysanthemi* strains (NCPPB 402, NCPPB 516 and NCPPB 3533), three *D. dadantii* strains (NCPPB 898, NCPPB 2976 and NCPPB 3537), one *D. paradisiaca* strain NCPPB 2511, eight *D. zeae* strains (ZJU1202 [15] and DZ2Q [16] isolated from diseased rice, CSL RW192 and MK19 isolated from river water, NCPPB 2538 isolated from maize, NCPPB 3531 and NCPPB 3532 isolated from potato, and MS1 isolated from banana [17]) and other five unassigned *Dickeya* spp. (NCPPB 569, NCPPB 3274, CSL RW240, DW0440 and MK17) [18]. These genome sequences may provide a good opportunity to study bacterial evolution and identify the genes contributing to host-range determination.

In this study, a complete genome sequence of *D. zeae* strain EC1, isolated from rice plants, was obtained using the Illumina next-generation sequencing technology coupling with polymerase chain reaction (PCR) method for gap closure. The genome sequence was annotated and compared with the representative genome sequences of other *Dickeya* species with a special focus on the virulence determinants and potential regulatory mechanisms. Substantial genomic rearrangements were revealed by comparing the genome sequence of *D. zeae* EC1 with other *Dickeya* isolates. Furthermore, we found that the gene cluster encoding for phytotoxin zeamine biosynthesis is conserved only in the *D. zeae* strains isolated from rice plants and the *D. solani* strains isolated from potato.

## Results and discussion

### General genomic features of *D. zeae* EC1

**Figure 1:**
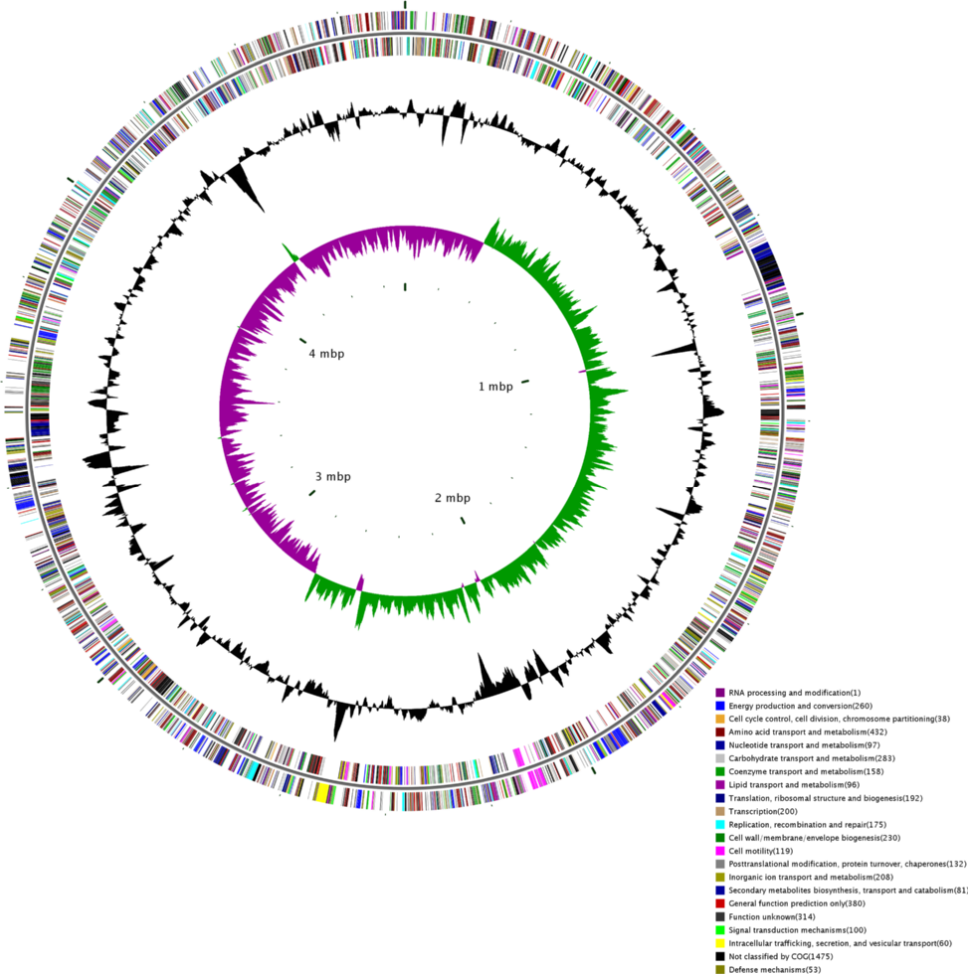
Circular chromosome map of *D. zeae* strain EC1. The distribution of genes is shown on the two outer rings. The next circle (black) indicates the GC content and the central circle (green/purple) shows GC-skew value. The window size of the GC content and GC-skew is 100 nt.

Assembly of *D. zeae* EC1 genome sequencing data resulted in 3 scaffolds with 3430.072 kb, 1294.767 kb and 1.821 kb in length, respectively, with 148× coverage in average. For finishing, the gaps were closed by custom primer walks or PCR amplification, followed by sequencing. Hence, the EC1 genome consists of a circular chromosome with 4,532,364 bps in size with no apparent autonomous plasmids. The average GC content of the whole genome is 53.43%. Coding sequences account for about 85.77% of the genome with GC content of 54.57%. The genome contains 4,154 open reading frames (ORFs) predicted to encode polypeptides with 935 bps in average length. The GC content in the intergenic region is 46.55%. An origin of replication designated as *dnaA* boxes was identified between the deduced gene for the 50S ribosomal protein L34 and the *gyrB* locus encoding DnaA, DnaN and RecF. The first nucleotide of the *dnaA* start codon was assigned as the base pair 1 of the chromosome (Table 1, Figure 1).

**Table 1:** **Genomic features of** ***D. zeae*** **EC1 and other** ***Dickeya*** **spp**

**Features**	**EC1**	**Ech1591**	**Ech586**	**3937**	**Ech703**
Size (bp)	4,532,364	4,813,854	4,818,394	4,922,802	4,679,450
GC content (%)	53.43	54.5	53.6	56.3	55
CDS					
Predicted no. of CDS	4,154	4,367	4,318	4,687	4,136
Function assigned	3,880	4,163	4,144	4,543	3,970
Function unknown	274	204	174	144	166
Ribosomal RNA operons	7	7	7	7	7
Transfer RNA	88	74	77	75	74
sRNA	10	3	3	14	4
Transposases	65	55	11	50	13

Seven rRNA operons were found in the chromosome of EC1 with 3 on the positive strand and 4 on the negative strand. Both chains contain an unusual rRNA operon with a 16S-23S-23S-5S organization, and another unusual one with 16S-23S-5S-5S organization locates on the negative chain, differing from the common 16S-23S-5S organization pattern. Similar unusual rRNA operons were also found in some other bacterial species, e.g. a 16S-23S-5S-5S gene cluster was found in *Erwinia pyrifoliae* Ep1/96, *E. tasmaniensis* Et1/99 [19]. The occurrence of the unusual organization of rRNAs within the chromosome was interpreted as the result of a chromosomal recombination event [19]. Altogether, 88 tRNA genes which recognize 20 codons were found in the genome of *D. zeae* EC1.

### Genome similarity of *D. zeae* EC1 and other Dickeya bacteria

The complete genomic sequences of several different *Dickeya* species including *D. chrysanthemi* Ech1591 (GenBank: CP001655.1; 4,813,854 bp), *D. zeae* Ech586 (GenBank: CP001836.1; 4,818,394 bp), *D. dadantii* 3937 (GenBank: CP002038.1; 4,922,802 bp) [11] and *D. paradisiaca* Ech703 (GenBank: CP001654.1; 4,679,450 bp) have been determined recently. In comparison, *D. zeae* EC1 has the smallest chromosome with 4,532,364 bp nucleotides with 4,154 ORFs, whereas *D. dadantii* 3937 possesses the largest genome with 4,922,802 bp nucleotides containing 4,687 ORFs (Table 1). The GC content of *D. zeae* EC1 genome is lowest (53.43%), similar to that of *D. zeae* Ech586 (53.6%) (Table 1). In addition, *D. zeae* EC1 contains substantially more tRNAs and transposases than any other bacterial species, suggesting a complex history of DNA transposition and recombination in the process of genome evolution [20]. Consistent with the above features, *D. zeae* EC1 has more ORFs encoding peptides with function unknown than any other *Dickeya* species with complete genome sequence (Table 1).

Phylogenetic relationships of *D. zeae* EC1 with other *Dickeya* species were assessed by performing Multilocus Sequence Analysis (MLSA) using four housekeeping genes (*atpD*, *dnaX*, *gyrB* and *recA*), and the concatenated data set of the four genes was constructed with *Pectobacterium atrosepticum* SCR1043 as an outgroup. The numbers of *Dickeya* species used in this phylogeny assessment were varied from 35 to 38 depending on the availability of the housekeeping gene sequences indicated above. The phylogenic analysis results showed that the three *D. zeae* strains isolated from diseased rice plants (EC1, ZJU1202 and DZ2Q) are located in the same branch, and share the same clade with the *D. zeae* isolates collected from other plants or sources (Additional file 1A, B, C, D). The same results also showed that among the four *Dickeya* strains with complete genome sequences released, EC1 was most homologous to *D. zeae* Ech586 and then *D. chrysanthemi* Ech1591 (Additional file 1A, B and C).

**Figure 2:**
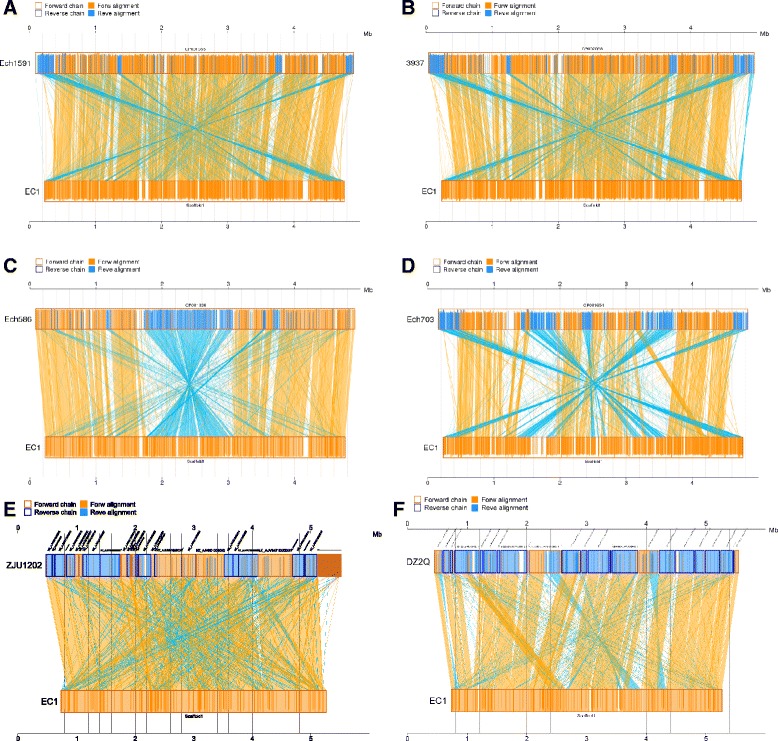
Nucleic acid colinearity of EC1 vs. Ech1591 **(A)**, 3937 **(B)**, Ech586 **(C)**, Ech703 **(D)**, ZJU1202 **(E)** and DZ2Q **(F)**, respectively. The sequence of EC1 is ordered according to that of the reference bacterium based on Mummer 3.22 (http://mummer.sourceforge.net/). Then upper and following axes of linear synteny graph are constructed after the same proportion of size reduction in length of both sequences (parameter: b, 200; c, 65; extend: l, 20). According to BLAST results, each pair nucleic acid sequence of two alignments is marked in the coordinate diagram according to its position information after the same proportion of size reduction. Orange lines connecting homologous regions present in the same orientation while the blues present inverted orientation.

**Figure 3:**
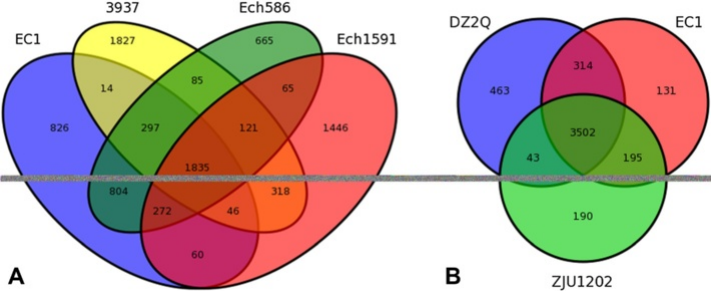
Venn diagrams for the deduced proteins of EC1, Ech1591, 3937, and Ech586 **(A)**, and the rice strains *D. zeae* EC1, ZJU1202 and DZ2Q **(B)**. Values were calculated by OrthoMCL clustering analyses using the following parameters: P-value Cut-off = 1 × 10^−5^; Identity Cut-off = 90%; Percent Match Cut-off = 80. The overlapping sections indicate shared numbers of deduced proteins.

To further evaluate the evolutionary relationships among the five *Dickeya* species with available complete genome sequences, the sequence of *D. zeae* EC1 was aligned with those of other four species by using BLAST program, respectively. The results showed that *D. zeae* EC1 is best co-linearly matched with *D. chrysanthemi* Ech1591 with a few rearrangement events (reverse match; blue) (Figure 2A), similarly, a few inversed fragments were observed between EC1 and *D. dadantii* 3937 (Figure 2B), while the alignment between EC1 and *D. zeae* Ech586 showed a big inversion with the largest amounts of homologous DNA fragments (Figure 2C). Intriguingly, the genes adjacent to the borders of this inversion are *W909_RS06895* and *W909_13075*, encoding an integrase IntB (WP_016943617.1) and a transposase (AJC68444.1), respectively, and the sequence between them is highly similar to that in Ech586 (*Dd586_2634* ~ *Dd586_1449*) with reverse orientation, suggesting that the inversion might be due to homologous recombination between these two genes. The entire length of the *D. paradisiaca* Ech703 genome was least matched with *D. zeae* EC1 with numerous gene content dissimilarities (Figure 2D). The distant relationship of *D. zeae* EC1 with other four complete *Dickeya* species indicated by the genomic collinearity analysis (Figure 2A-D) is supported by the estimated numbers of shared genes through OrthoMCL comparative analyses (only 1835 proteins in total, Figure 3A). The number of the shared genes between *D. zeae* EC1 and *D. chrysanthemi* Ech1591, *D. zeae* Ech586 and *D. dadantii* 3937 was 2213, 3208 and 2192, respectively (Figure 3A). Agreeable with the genomic collinearity analysis shown in Figure 2, the most dissimilar species *D. paradisiaca* Ech703 shared only 961 genes with *D. zeae* EC1, and 935, 964 and 940 with *D. chrysanthemi* Ech1591, *D. zeae* Ech586, and *D. dadantii* 3937, respectively.

To identify the genes specific to *D. zeae* EC1, we compared its genome sequence to the released complete genome sequences of *D. chrysanthemi* Ech1591, *D. zeae* Ech586, *D. paradisiaca* Ech703 and *D. dadantii* 3937, which unveiled 826 unique proteins located only in the EC1 genome (Figure 3A). For example, the chromosome of EC1 harbors several large specific regions, including the region from 1418747 nt to 1469974 nt, involved in the biosynthesis of zeamine phytotoxins, containing two characterized zeamine synthase genes *zmsA* and *zmsK* [6,7], the region from 1551137 nt to 1566214 nt, encoding different peptide products including three HTH-type transcriptional regulators (AJC65834.1, AJC65839.1 and AJC65846.1), three transporter proteins (AJC65835.1, AJC65838.1, and AJC65845.1), an acetyltransferase (AJC65836.1), a phosphoserine phosphatase (AJC65837.1), an acetaldehyde dehydrogenase (AJC65840.1), a transposase IS4 (AJC64674.1), a chemotaxis protein CheX (AJC65842.1), and five hypothetical proteins (AJC68379.1, AJC68380.1, AJC68381.1, AJC65843.1 and AJC68382.1) .

Taken together, the above analyses show that *D. zeae* EC1 is most co-linear with *D. chrysanthemi* Ech1591, most conserved with *D. zeae* Ech586 and least similar to *D. paradisiaca* Ech703. The findings highlight substantial divergence among *Dickeya* species. For paving ways to understand the genetic basis of pathogenicity and host specificity, we specifically compared the similarity and divergence of the genes encoding virulence determinants and regulatory mechanisms in the following sections.

In addition, we compared the genome sequences of three *D. zeae* strains isolated from rice plants. Synteny analysis showed that EC1 isolated from Guangdong Province of China was 99.962% identity to strain ZJU1202, which was also from Guangdong Province of China [15], and 95.863% identity to strain DZ2Q from Italy [16] (Figure 2E, 2 F), and the number of the shared genes among these three rice pathogen strains is up to 3502, only 131 genes are unique in strain EC1 (Figure 3B), among which, 95.4% (125 out of 131) encode hypothetical proteins, and the 6 genes with known functions share high similarities with other *Dickeya* strains (Additional file 2), 4 of which encode tranposases (*457403* ~ *457603 nt*, AJC65190.1, AJC65197.1, and AJC65071.1), 1 encode citrate lyase acyl carrier protein CitD (AJC65916.1), and the remaining encodes a PilT plasmid maintenance protein pirin (AJC64970.1), respectively. Given the high level of genome sequence similarity and the fact that the genomes of strains ZJU1202 and DZ2Q have only been partially sequenced with numerous gaps, which hinders the accurate comparison, we believe that the common genes shared by these three strains could be substantially higher than the currently obtained 3502.

### Cell wall-degrading extracellular enzymes and proteases

Extracellular enzymes, including pectinases, polygalacturonases, cellulases and proteases, are one of the key virulence factors during bacterial pathogenesis when infecting host plants to cause soft rot symptoms. Among these enzymes, pectate lyases (Pel) and other pectinases such as pectin methylesterases (Pem) and pectin lyases (Pnl) of *D. dadantii* 3937 have been shown to play a major role in the virulence and tissue maceration [21]. The genome of EC1 contains a total of 14 genes encoding pectin degradation enzymes, including *pnl*, *pelN*, *pelL*, *pelI*, *pelA*, *pelE*, *pelD*, *pelC*, *pelB*, and *pelZ* encoding pectate lyases, *pelW* and *pelX* encoding pectate disaccharide-lyases, *paeY* encoding a pectin acetylesterase, and *pemA* encoding a pectin methylesterase (Table 2). These pectin degradation genes are highly conserved in various *Dickeya* species, except that *pnl* is absent in *D. zeae* Ech586 and *D. chrysanthemi* Ech1591, and *pelI* and *pelD* are not included in the *D. paradisiaca* Ech703 genome (Table 2). The *pehK* and *pehX* genes encoding polygalacturonases are also conserved in *Dickeya* spp. except that *pehX* has two copies in *D. chrysanthemi* Ech1591 (*pehW* and *pehX*), and three copies in *D. dadantii* 3937 (*pehV*, *pehW* and *pehX*) (Table 2).

**Table 2:** **Homolog of cell wall-degrading enzyme genes in** ***D. zeae*** **EC1 and other** ***Dickeya*** **spp**

Genes in EC1*	**Accesion no. in EC1**	**Ech586**		**Ech1591**		**3937**		**Ech703**	
		**Accesion no.**	**Homology (%)**	**Accesion no.**	**Homology (%)**	**Accesion no.**	**Homology (%)**	**Accesion no.**	**Homology (%)**
***pnl***	AJC65107.1	N/A	N/A	N/A	N/A	ADM96804.1	95	ACS87140.1	89
***pelN***	AJC66160.1	ACZ77096.1	97	ACT07147.1	92	ADM98300.1	91	ACS85991.1	79
***pelL***	AJC66869.1	ACZ76356.1	97	ACT06383.1	92	ADM99100.1	94	ACS85679.1	80
***pelI***	AJC67145.1	ACZ77772.1	98	ACT06136.1	85	ADM99410.1	94	N/A	N/A
***pelA***	AJC67288.1	ACZ77918.1	98	ACT05963.1	91	ADM99550.1	92	ACS86540.1	70
***pelE***	AJC67289.1	ACZ77919.1	96	ACT05962.1	90	ADM99551.1	93	ACS86541.1	71
***pelD***	AJC67290.1	ACZ77920.1	80	ACT05961.1	78	ADM99552.1	84	N/A	N/A
***pelC***	AJC67296.1	ACZ78618.1	99	ACT05163.1	94	ADN00345.1	96	ACS84167.1	87
***pelB***	AJC67297.1	ACZ78619.1	98	ACT05162.1	93	ADN00346.1	94	ACS84166.1	89
***pelZ***	AJC67298.1	ACZ78620.1	96	ACT05161.1	93	ADN00347.1	93	ACS84165.1	84
***pelW***	AJC66425.1	ACZ76819.1	98	ACT06819.1	91	ADM98617.1	90	ACS85823.1	83
***pelX***	AJC68275.1	ACZ78987.1	97	ACT08992.1	92	ADN00761.1	92	ACS87748.1	79
***paeY***	AJC67291.1	ACZ77921.1	93	ACT05960.1	89	ADM99553.1	84	ACS86542.1	72
***pemA***	AJC67292.1	ACZ77922.1	98	ACT05959.1	93	ADM99554.1	93	ACS86543.1	79
***pehK***	AJC67475.1	ACZ78152.1	97	ACT05703.1	86	ADM99816.1	86	ACS84778.1	60
***pehV***	N/A	N/A	N/A	N/A	N/A	ADN00465.1	69	N/A	N/A
***pehW***	N/A	N/A	N/A	ACT05046.1	77	ADN00466.1	83	N/A	N/A
***pehX***	AJC68058.1	ACZ78734.1	96	ACT05045.1	90	ADN00467.1	89	ACS84017.1	76
***celZ***	AJC66868.1	ACZ76357.1	92	ACT06384.1	79	ADM99099.1	80	ACS85678.1	65
***celY***	AJC68171.1	ACZ78883.1	98	ACT08877.1	88	ADM96331.1	87	ACS87644.1	76
***bglA***	AJC64939.1	ACZ75273.1	98	ACT08535.1	94	ADM96608.1	94	N/A	N/A
***bgxA***	AJC65979.1	ACZ77342.1	98	ACT07381.1	94	ADM98002.1	93	ACS86228.1	79
***bglB***	AJC66701.1	ACZ76526.1	97	ACT06558.1	95	ADM98908.1	94	ACS85465.1	84
***nagZ***	AJC66758.1	ACZ76466.1	98	ACT06490.1	93	ADM98979.1	93	ACS85403.1	82
***bglC***	AJC67553.1	N/A	N/A	ACT05629.1	88	ADM99901.1	89	ACS84576.1	92
***bglD***	AJC67554.1	N/A	N/A	ACT05628.18	95	ADM99899.1	94	ACS84575.1	93
***celH***	AJC67562.1	ACZ78240.1	98	ACT05617.1	96	ADM99910.1	95	ACS84568.1	89
***lfaA***	AJC66752.1	ACZ76472.1	*98*	ACT06496.1	94	ADM98973.1	92	N/A	N/A
***prtX***	AJC66333.1	ACZ76915.1	98	ACT06956.1	91	ADM98496.1	90	N/A	N/A
***prtC***	AJC66334.1	ACZ76914.1	98	ACT06955.1	86	ADM98497.1	87	N/A	N/A
***prtB***	AJC66335.1	ACZ76913.1	96	ACT06954.1	90	ADM98498.1	91	N/A	N/A
***prtG***	AJC66340.1	ACZ76908.1	97	N/A	N/A	ADM98503.1	90	N/A	N/A

*AJC65107.1, AJC66160.1*,* AJC66869.1, AJC67145.1, AJC67288.1, AJC67289.1, AJC67290.1, AJC67296.1, AJC67297.1, and AJC67298.1 are pectate lyases, AJC66425.1 and AJC68275.1 are pectate disaccharide-lyases, AJC67291.1 is a pectin acetylesterase, and AJC67292.1is a pectin methylesterase.

Similarly, ten genes in EC1 are involved in cellulose degradation, including 2 endoglucanase encoding genes *celY* and *celZ*, 7 beta-glucosidase encoding genes *bglA*, *bgxA*, *bglB*, *nagZ*, *bglC*, *bglD* and *celH*, and an alpha-glucosidase encoding gene *lfaA*. These genes, associated with oligosaccharide degradation, are conserved in *Dickeya* stains, except that *bglC* and *bglD* were absent in *D. zeae* Ech586, and *bglA* and *lfaA* were not found in *D. paradisiaca* Ech703 (Table 2).

The *prtGDEFBCX* cluster (*W909_09760 ~ 09795*) encoding four proteases and three protease secretion associated proteins is located on the negative strand of EC1 chromosome. Among them, the four protease encoding genes, i.e., *prtG*, *B*, *C* and *X,* encode serralysin homologs sharing about 59.8% similarity at amino acid level, and the *prtD, E* and *F* encode the type I secretion system (T1SS) components PrtDEF (AJC66336.1, AJC66336.1, and AJC66336.1). In addition, a proteinase inhibitor encoding gene *inh* is located between *prtG* and *prtD*. Alignment with other *Dickeya* species showed that *prtG* is absent in Ech1591, while the whole *prt* gene cluster is lost in the genome of *D. paradisiaca* Ech703 (Table 2).

### Type II secretion system

The type II secretion system (T2SS) is used by diverse gram-negative bacteria to translocate extracellular proteins across the outer membrane. In *D. dadantii* 3937, the T2SS is encoded by the *out* genes and *stt* genes. The Out system allows the secretion of several proteins including most pectinases while the Stt system only presents to the outer part of the outer membrane, such as the pectin lyases encoded by the adjacent *pnl* gene [22,23]. *D. zeae* EC1 chromosome contains a highly conserved T2SS gene cluster (*outSBCDEFGHIJKLMO*; *W909_13825 ~ 13895*) (Additional file 3), covering 13.814 kb with 15 ORFs, but no *stt* genes. The gene cluster shares a high similarity with that of *D. zeae* Ech586 (coverage 100%, identity 94%), *D. chrysanthemi* Ech1591 (coverage 90%, identity 87%), *D. dadantii* 3937 (coverage 89%, identity 85%), and *D. paradisiaca* Ech703 (coverage 77%, identity 77%) at nucleic acid level. The *out* gene cluster is highly conserved among *Dickeya* spp. except that *D. zeae* EC1 harbors an extra predicted gene designated as *W909_13885* encoding a hypothetical protein (AJC68452.1) at the upstream of *outC*, and that *D. dadantii* 3937 contains an additional *outT* gene (Additional file 3).

### Type III secretion system encoded by the *hrp* gene cluster

**Figure 4:**
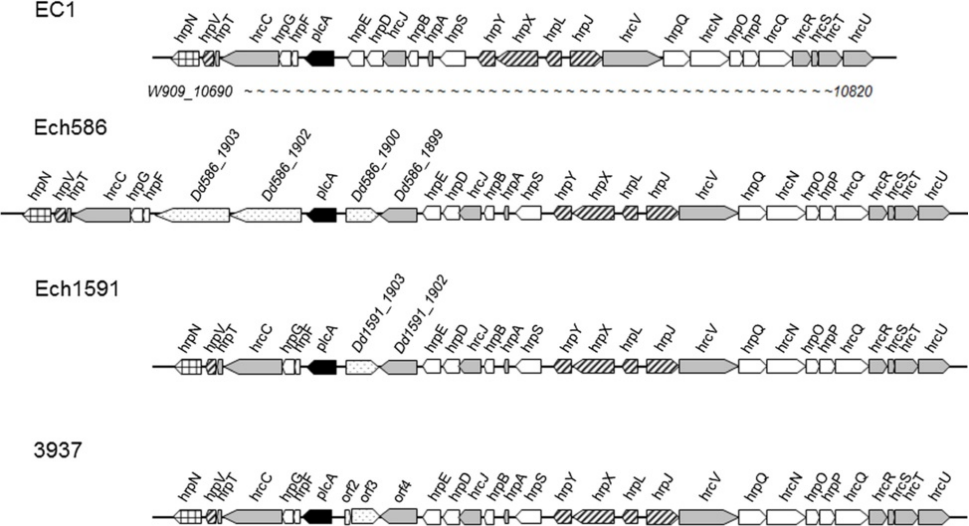
Genetic organization of the *hrp* cluster in *Dickeya* spp. The varied genes are indicated by filled arrows. Symbol: , *hrp* response elicitor gene; , regulator gene; , T3SS structural gene; , hypothetical gene; , extracellular phospholipase gene *plcA*. The *hrp* genes are fully conserved except the genes adjacent to *plcA*.

The hypersensitive response and pathogenicity (Hrp) type III secretion systems (T3SS) were established as pathogenicity factors in many phytopathogenic bacteria [24,25]. Similarly, the *hrp* genes in *Dickeya* spp. have also been reported to play an important role in their pathogenesis and interaction with host plants [26-28]. In the genome of *D. zeae* EC1, a large *hrp* gene cluster spanning 24.8 kb was identified, which is composed of 27 genes and arranged in three transcriptional units. Alignment of this gene cluster with the counterparts in other *Dickeya* species showed that this *hrp* gene cluster is absent in *D. paradisiaca* Ech703, but present in other three species, i.e., *D. chrysanthemi* Ech1591, *D. zeae* Ech586, and *D. dadantii* 3937 with certain variations (Figure 4). Among the strains containing the *hrp* gene cluster, the only variation was found at the adjacent regions of the *plcA* gene, which encodes an extracellular phospholipase [29]. The *plcA* gene is located between *hrpE* and *hrpF* genes, in *D. zeae* EC1, there is no additional genes, while in Ech586, *Dd586_1900* (encoding a hypothetical protein) and *Dd586_1899* (encoding a lytic murein transglycosylase) are located at the upstream, and *Dd586_1902* and *Dd586_1903* (both encoding hypothetical proteins) are located at the downstream (Figure 4). Similarly, *Dd1591_1903* and *Dd1591_1902* (encoding a HrpE/YscL family type III secretion apparatus protein) are located at the upstream in *D. chrysanthemi* Ech1591, and in *D. dadantii* 3937, three ORFs encoding two hypothetical proteins and a membrane-bound lytic murein transglycosylase MltB, respectively are located at the upstream (Figure 4, additional file 4). However, the biological significance of these extra genes surrounding *plcA* has not yet been investigated.

Some of these *T3SS* genes of the *Dickeya* species have been characterized in details. Among them, *hrpXY* encode a two-component system (TCS), which is required for the activation of the *hrp* gene expression in *D. dadantii* 3937 [30]. After phosphorylation, HrpY binds to the *hrpS* promoter and activates the expression of *hrpS*, which encodes a sigma (54)-dependent enhancer-binding protein HrpS. In turn, HrpS binds to the sigma factor homolog *hrpL*, and consequently activates the genes encoding T3SS structural proteins such as HrcJ and HrpA and secreted effectors including *hrpN* [30]. In *D. dadantii* EC16, the Hrp system appears to contribute to an early stage of pathogenesis, whereas in *D. dadantii* 3937 mutation of *hrpN*, *hrpG,* and *hrcC* resulted in substantially reduced lesion formation and bacterial growth in African violet leaves [26,27]. In addition to playing a role in plant–microbe interactions, the *D. dadantii* T3SS is also required for the formation of bacterial aggregates at the air-liquid interface [30,31]. Given that most key *hrp* genes are highly conserved in *D. zeae* EC1, it is highly possible that the T3SS in *D. zeae* EC1 could also play certain roles in the bacterial virulence and physiology, which awaits further characterizations.

### Type IV secretion system

Type IV secretion system (T4SS) is unique from the other secretion systems in its ability to translocate nucleic acids in addition to proteins from donor to recipient cells [32]. It functions in conjugation, pathogenicity and DNA release/uptake [32,33]. In the EC1 genome, T4SS gene cluster covers 9.742 kb, harboring 11 T4SS-core genes (*W909_12990 ~ 13040*) encoding VirB1 ~ 11 (AJC66938.1 *~* AJC66948.1) (Additional file 5). VirB1 forms bores hole in peptidoglycan layer allowing T4SS complex assembly to occur, while previous studies indicated that VirB2 and VirB5 proteins constitute an extracellular pilus of T4SS, *virB4* and *virB11* encode ATPases providing energy and motor force for macromolecular secretion, architecture assembly and substrate pumping, and VirB6, VirB8, and VirB10 form a membrane channel encompassing both membranes, and the periplasmic protein VirB9 in complex with the short lipoprotein VirB7 could be part of this structure [32,34,35]. This T4SS gene cluster is highly conserved in *D. chrysanthemi* Ech1591 (coverage 100%, identity 97%), *D. zeae* Ech586 (coverage 99%, identity 96%), and *D. dadantii* 3937 (coverage 100%, identity 96%), but not found in *D. paradisiaca* Ech703.

### Type VI secretion system

**Figure 5:**
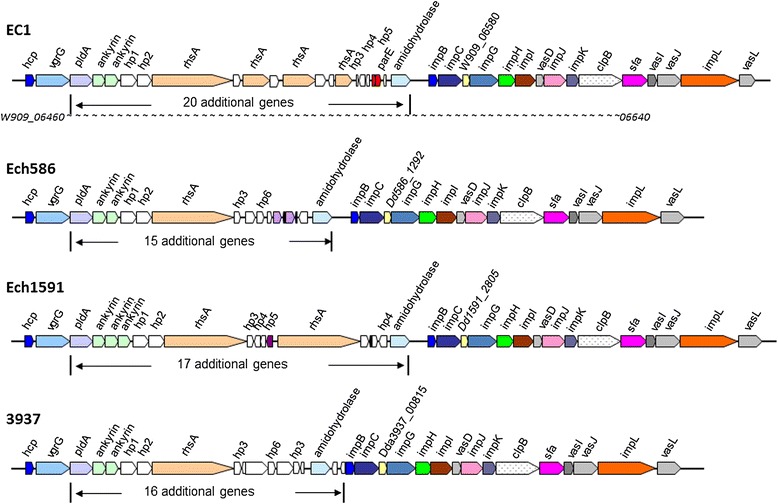
Genetic organization of the T6SS gene cluster in *Dickeya* spp. The variable region is indicated under the gene cluster. Colored ORF indicates the genes with known function, and the same color represents the same biological function. The gene encoding hypothetical protein is indicated by open ORF.

The type VI secretion system (T6SS) was identified recently and initially thought to take part in bacterial pathogenicity and host colonization [36]. Subsequent studies showed that T6SS functions in various biological processes, such as mediating cooperative or competitive interactions between bacteria and eukaryotes, and bacterial biofilm formation [37-41]. T6SS is typically encoded by clusters of 12 to 20 genes, with a minimum of 13 genes for production of a functional apparatus [42]. In *Dickeya* genus, *D. zeae* Ech586 and *D. chrysanthemi* Ech1591 were shown to possess an identical T6SS loci consisting of 17 genes [43], but the biological function has not yet been determined. In this study, a gene cluster encoding T6SS was found in *D. zeae* EC1, spanning 44.774 kb with 37 ORFs designated from *W909_06460* to *W909_06640*. In this gene cluster, in addition to the 17 T6SS genes described previously [43], there are 20 additional ORFs inserted between *W909_06465* (*vgrG* encoding Valine-glycine repeat G) and *W909_06570* (*impB*) genes (Figure 5). These inserted genes encode a PAAR repeat-containing protein phospholipase A1 PldA (AJC65737.1), two ankyrins (AJC65738.1 and AJC65739.1), four YD repeat-containing protein RhsAs (AJC65742.1, AJC65744.1, W909_06510 and AJC65746.1), two plasmid stabilization system protein ParEs (AJC65749.1 and AJC65750.1), a peptidase M20 (AJC65751.1) and 10 hypothetical proteins (AJC65740.1, AJC65741.1, AJC65743.1, AJC68373.1, AJC68374.1, AJC65745.1, AJC65747.1, AJC65748.1, W909_06545, and AJC68375.1), amongst, AJC68373.1 and AJC68374.1 were not found in any other *Dickeya* strains. The 17 core T6SS genes are highly conserved in *D. dadantii* 3937, *D. zeae* Ech586 and *D. chrysanthemi* Ech1591, but substantial variations were found in the additional genes inserted between *vgrG* and *impB* (Figure 5). Similar to the cases of T3SS and T4SS, the T6SS gene cluster is totally absent in the genome of *D. paradisiaca* Ech703.

### Flagellar and chemotaxis genes

One set of gene cluster encoding flagellar biosynthesis and chemotaxis proteins was found in the genome of *D. zeae* EC1 (*W909_12260 ~ 12570*), covering 63.371 kb in length. The gene cluster encodes 40 proteins (FliZ, FliA, 2 FliCs, FliD *~* T, FlgA *~* N, and FlhA *~* E) involved in flagellar biosynthesis, 10 chemotaxis-associated proteins (CheZ, CheY, CheB, CheR, MCP1, CheW, CheA, MotB, MotA, and MCP3) (Additional file 6), two methyltransferases (AJC66805.1 and AJC66816.1), an aminotransferase (AJC66815.1), and 9 fatty acid biosynthesis proteins (*W909_12275 ~ 12315*) (Additional file 7). The gene cluster closely resembles that found in *D. zeae* Ech586 (coverage 83%, identity 93%), *D. chrysanthemi* Ech1591 (coverage 82%, identity 83%), *D. dadantii* 3937 (coverage 83%, identity 84%) and *D. paradisiaca* Ech703 (coverage 49%, identity 77%) in nucleotide sequence, except that *D. zeae* EC1 contains a big inserted fragment designated as *W909_12275 ~ 12315* encoding fatty acid biosynthesis (Additional file 6; Additional file 7). A blast search found that these *D. zeae* EC1 specific genes encoding an acyl-CoA reductase (AJC66806.1), a long-chain-fatty-acid--luciferin-component ligase LuxE (AJC66807.1), a long-chain acyl-CoA synthetase FadD (AJC66808.1), two transketolases TktA (AJC66809.1) and TktB (AJC66810.1), and two 3-oxoacyl-[acyl-carrier protein] reductases FabG (AJC66811.1 and AJC66812.1), an acyl carrier protein (AJC66813.1) and a maltose O-acetyltransferase (AJC66814.1), respectively, which share 47-70% protein similarity to their homologs in *Pantoea ananatis* and seem unlikely associated with the flagellar biogenesis. This fragment was also found between the fragellar genes in the rice strains DZZ2Q and ZJU1202 in same gene organization, but absent in other sequenced *D. zeae* strains. The flagellar genes are not clustered in the genome of *D. paradisiaca* Ech703, indicating again its distant evolution relationship with the other four *Dickeya* strains.

Unlike some other *Enterobacteriaceae*, which encode more than one type of flagella and flagellin [44], *Dickeya* spp. encodes only one type of flagella and one type of flagellin. Flagellar proteins are generally responsible for cell motility and intracellular trafficking, secretion and vesicular transport, while the chemotaxis proteins are responsible for cell motility and signal transduction. In *D. dadantii* 3937, mutation of *fliA* encoding a sigma factor abolished the bacterial motility, significantly reduced Pel production and the bacterial attachment to plant tissues, demonstrating that FliA is a positive regulator of many traits associated with virulence [44]. Mutation of chemotactic genes (*cheW*, *cheB*, *cheY* and *cheZ*) caused substantial reduction in swimming motility and decreased the bacterial virulence against *Arabidopsis* and potato [45]. The roles of flagellar and chemotaxis systems in *D. zeae* infection, especially in pathogen-monocotyledon host interactions, remain to be investigated.

### Quorum-sensing systems

Many Gram-negative bacterial pathogens utilize the *luxI/luxR* quorum sensing system to regulate the expression of virulence genes. Typically, *luxI* encodes a synthase for production of acylhomoserine lactone (AHL) family quorum sensing signals, and *luxR* encodes an AHL signal receptor. Upon interaction with AHL signal, LuxR becomes an active transcription factor and hence modulating the expression of virulence genes. Our previous study showed that *D. zeae* EC1 produces an AHL family quorum sensing signal, i.e., *N*-(3-oxo-hexanoyl)-homoserine lactone (OHHL), which is encoded by the *luxI* homolog *expI* [8]. Mutation of *expI* resulted in the increased bacterial cell motility and decreased biofilm formation [8]. A Blast search of the EC1 genome revealed only one copy of *expI* (*W909_00485*) and also one well-conserved *luxR* homolog (*W909_00480*) designated as *expR*. ExpR is highly conserved in *D. zeae* Ech586 (97%), 3937 (91%), and *D. chrysanthemi* Ech1591 (91%), and *D. paradisiaca* Ech703 (64%). Interestingly, ExpI is absent in *D. paradisiaca* Ech703 (Additional file 8).

AI-2 produced by the S-ribosylhomocysteine lyase LuxS represents another type of conversed QS system involved in bacterial interspecies communication [46]. In *Dickeya* spp., evidence suggests that AI-2 production was switched off by indole-3-acetic acid (IAA) pathway under tryptophan control [47], but the biological significance of tryptophan modulation in planta and the role of AI-2 in pathogenesis remains unclear. A *luxS* gene homolog (*W909_04510*) encoding a S-ribosylhomocysteine lyase was also identified in *D. zeae* EC1, sharing about 79% identity to the *luxS* gene of *Serratia marcescens* ATCC 274 [48], and about 79% - 93% identity to its homologs in Ech586, Ech1591 and Ech703 at nucleic acid level, respectively.

Interestingly, in the upstream of the *expI/expR* genes (*W909_00480 ~ 00485*), the *vfm* gene cluster encoding the biosynthesis of a novel QS signal was found highly conserved in *D. zeae* EC1 and other four *Dickeya* species and strains used in this study (70% to 98% identity at nucleic acid level, additional file 9, Additional file 10). The gene cluster was originally identified in *D. dadantii* 3937, and is associated with the regulation of virulence factor production and pathogenesis [49]. The biological significance of various QS systems in *D. zeae* EC1 and other *Dickaya* species remains to be further investigated.

### Zeamine synthesis gene cluster

**Figure 6:**
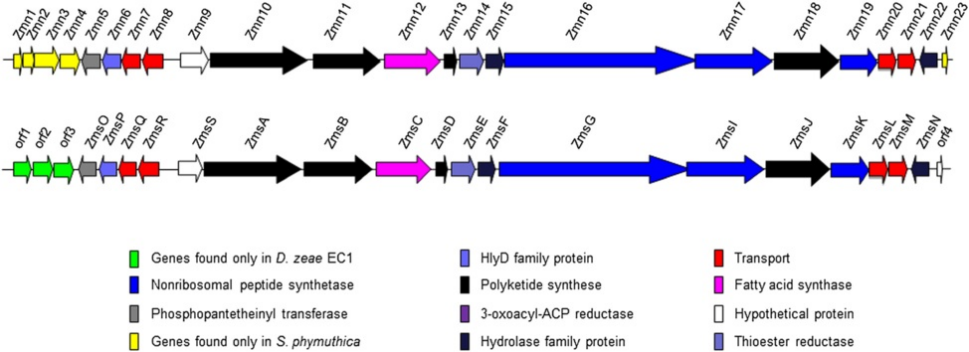
Genetic organization of zeamine biosynthetic gene cluster in *S. phymuthica* AS12 (top), *D. zeae* EC1 and *D. solani* D s0432-1 (bottom).

*D. zeae* EC1 produces at least two polyamino phytotoxins and antibiotics, i.e., zeamine and zeamine II, which were shown to be the major virulence determinants [6]. The genes *zmsA* and *zmsK,* which respectively encode a multidomain polyketide synthase and an non-ribosomal peptide synthase containing only a condensation domain, are involved in zeamines biosynthesis and play a key role in the bacterial virulence [6,7]. Interestingly, zeamines have also been identified from a bacterial isolate of *Serratia plymuthica* RVH1 and a gene cluster containing 23 ORFs was proposed as the zeamine biosynthetic gene (*zmn*) cluster [50]. As the genome and the *zmn* sequences of *S. plymuthica* RVH1 are not publically available, we used the same gene cluster from *S. plymuthica* AS12 for comparison analysis. We found that most of the *zmn* genes of *S. plymuthica* are conserved in the *D. zeae* EC1 genome (*W909_06685 ~ 06770*) except a few genes including *zmn1-4* and *zmn23* (Figure 6, additional file 11). Moreover, as expected, the two sets of zeamine biosynthetic genes from *D. zeae* EC1 and *S. plymuthica* AS12 share a high degree of similarity with amino acids identity ranging from 50-92% (Additional file 11). *S. plymuthica* is an uncommon cause of human infection, but the role of zeamines in its pathogenesis has not yet been determined

The *zms* gene cluster of *D. zeae* EC1 spans 51,169 bp, and includes 18 ORFs ranging from *zmsO* to *zmsN* (Figure 6). Surprisingly, the *zms* gene cluster is not conserved in the four *Dickeya* strains used in this study. Alignments of the nucleic acid sequence of the *zms* gene cluster showed that only few sporadic fragments in the cluster are present in the four whole-genome sequenced *Dickeya* spp., but surprisingly, it shares 69.35% similarity (95% coverage, 73% identity) with that in *D. solani* strains, such as 3337, D s0432-1, MK10, MK16, M005 and GBCC. The zeamine biosynthetic genes from *D. zeae* EC1 and *D. solani* D s0432-1 share a high degree of similarity with amino acids identity ranging from 59-94% (Additional file 11). We have also searched the partial genome sequences of other nine *D. zeae* strains, and found surprisingly that this *zms* gene cluster is only fully or almost fully conserved in the rice pathogens ZJU1202 [15] and DZ2Q [16] with 100% and 99% identity at nucleotide level, but absent in other *D. zeae* strains isolated from river water, maize, potato and banana, respectively [17,18]. Noticeably, the GC content of this cluster from *D. zeae* EC1 is 48.77%, significantly lower than the genomic GC contents of strains EC1 (53.43%), Ech1591 (54.5%), Ech586 (53.6%), Ech703 (55%) and 3937 (56.3%), suggesting that this zeamine biosynthesis gene cluster was acquired through horizontal gene transfer from other organisms at a relatively later stage of bacterial evolution, and seemingly associated with host specificity.

## Conclusion

In this study, we present a complete genome sequence of *D. zeae* strain EC1 isolated from rice plants, which together with the other four released complete genome sequences of *Dickeya* species, i.e., *D. chrysanthemi* Ech1591, *D. zeae* Ech586, *D. dadantii* 3937, and *D. paradisiaca* Ech703, allows detailed genomic comparison to study bacterial evolution and identify clues associated with pathogenesis and host specificity. Synteny analysis of *D. zeae* EC1 and the other four released *Dickeya* spp. showed that the genome sequences of *D. zeae* strain EC1 is most co-linear with *D. chrysanthemi* Ech1591, most conserved with *D. zeae* Ech586 and least similar to *D. paradisiaca* Ech703. This is also evident from the overall similarity of the virulence genes conserved in the *Dickeya* strains used in this study. Several groups of virulence genes, such as protease genes (Table 2), T1SS gene cluster *prtDEF*, T3SS gene cluster (Figure 4), T4SS gene cluster (Additional file 5) and T6SS gene cluster (Figure 5) are highly or fully conserved in *D. zeae* EC1 and *D. zeae* Ech586 but absent in the genome of *D. paradisiaca* Ech703.

Except for *D. paradisiaca* Ech703, most virulence genes are well-conserved in other four *Dickeya* pathogens. However, we also noted some variations in certain virulence determinants, for example, the T3SS (*hrp)* and T6SS gene clusters. Given the important role of these two virulence determinants in host-pathogen interactions, it would be interesting to determine the impact of such variations on bacterial virulence and host-specificity. Importantly, a range of unique genes associated with virulence were identified in *D. zeae* strain EC1, such as zeamine synthesis gene cluster, 4 hypothetical proteins in the T6SS gene cluster (AJC65743.1, AJC68373.1, AJC68374.1 and AJC65745.1), and the 9 fatty acid biosynthesis proteins (AJC66806.1 ~ AJC66814.1) in the flagella biosynthesis cluster (Additional file 7). Among them, the genes encoding zeamine biosynthesis have been shown critical for *D. zeae* EC1 to establish infections in rice [6,7]. Intriguingly, the *zms* gene cluster was found in genomes of the six sequenced *D. solani* strains and strains of *S. plymuthica* RVH1 and AS12, suggesting that this cluster is possibly derived from a same organism by horizontal gene transfer during bacterial evolution. Given that the high similarity of the proteins encoded by the fragment inserted in the flagellar cluster (*W909_12275 ~ 12315*)) to *P. ananatis* (Additional file 7), we were tempted to speculate that this gene fragment may be transferred from an unknown organism or similar organisms to *D. zeae* EC1, *P. ananatis* strains AJ13355 (isolated from soil in Japan) and LMG20103 (isolated from Eucalyptus blight and dieback in South Africa).

In the last few years, up to 8 *D. zeae* isolates have been partially sequenced. Most intriguingly, sequence alignment showed that the *zms* gene cluster was only found in the *D. zeae* strains EC1, ZJU1202 and DZ2Q infecting rice plants [8,15,16], but not in other *D. zeae* strains from environment or other hosts, such as CSL RW192 and MK19 isolated from river water, NCPPB 2538 isolated from maize [51], Ech586 isolated from *Philodendron* [10], NCPPB 3531 and NCPPB 3532 isolated from potato, and MS1 isolated from banana [17,18]. Phylogenetic analysis of *D. zeae* strains indicates that these *D. zeae* strains are closely related. In particular, three rice isolates were found in the same evolutionary clade (Additional file 1). Taken together, our data suggests that the remaining *D. zeae* strains can be at least divided into two pathovars with one infecting rice and the other infecting other crops. We proposed to reclassify these rice strains as *D. zeae* subsp. *oryzae*.

In summary, comparison of the genome information of *D. zeae* EC1 with the closely related *Dickeya* species provides new insights for the conservation and evolution processes of virulence determinants in these important bacterial pathogens. The overall similarity in extracellular enzymes and *hrp* and T6SS systems of EC1 and other *Dickeya* pathogens seems to explain well why EC1 could also infect dicotyledons, and the unique genes (*W909_06510* and *W909_06520*) in T6SS gene clusters might hold the keys to decipher the mechanisms of EC1 in infecting monocotyledons. Experimental dissection of the roles of these strain-specific genes and variations identified in this study shall reveal novel insights into the molecular mechanisms that specify host ranges and pathogenesis.

## Methods

### Whole-genome sequencing

Genomic DNA was extracted from *D. zeae* EC1 grown at 28°C in YEB medium [8] using MasterPure DNA purification kit (EPICENTRE Biotechnologies). The complete genome sequence was determined by the Beijing Genome Institute (Shenjun, China) using Solexa technology. A shotgun paired-end library with a fragment size between 150 to 500 bp and a long jumping distance mate-pair library with an insert-size between 2 to 10 kb were constructed. The two libraries were sequenced, and reads were trimmed on quality and length. In total, 1,664,407 high-quality filtered sequence reads were generated, with an average coverage equivalent to 148×. Sequence assembly was carried out using the SOAP software (http://soap.genomics.org.cn) 7 with a read length of 0.5 and a similarity of 0.8. Seven contigs were generated and joined into 3 scaffolds using pair-end information, with 3430.072 kb, 1294.767 kb and 1.821 kb in length, respectively. The *in silico* finishing of some gaps was carried out by mapping. We used the borders of the gaps as anchor and retrieved the reads in both orientations to perform a new *de novo* assembly on the gaps. The mapped reads were collected and used for *de novo* local assembling (read length of 0.5 and similarity of 0.8). Other gaps were closed by PCR amplification followed by DNA sequencing. The genome sequence data has been deposited in NCBI database with accession number CP006929.1.

### Gene prediction and annotation

Genes were predicted using the coding sequence (CDS) prediction program Glimmer 3.0 [52]. Amino acid genome comparison was performed by bi-directional BLASTp (2.2.21) sequence alignment of translated ORFs in the Nr, Nt, SwissProt, COG (release: 20090331), and KEGG databases with a 10^−5^ e-value threshold. The best hit was filtered using a 50% identity cut-off value. Genes were considered as strain-specific if identity of the encoded protein was lower than 80% on the similar portion between them. In addition, ORFs with a size less than 110 bp were cut-off by Glimmer 3.0. Transposon sequences were searched using the RepeatMasker and RepeatProteinMasker softwares. Tandem repeat sequences were predicted by Tandem repeat finder (TRF) and aligned in the known transposon sequence database. rRNAs were found in rRNA database or predicted by rRNAmmer [53], tRNAscan [54] was used to predict the tRNA regions and analyze the tRNA secondary structures, and Rfam was used to analyze miRNA, sRNA and snRNA.

### Phylogenetic analysis

Relationships of EC1 with other isolates of *Dickeya* were determined from multilocus sequence analysis (MLSA) on four housekeeping genes including *atpD*, *dnaX, gyrB* and *recA*, which were always used for the identification of *Dickeya* spp.[1,55]. *AtpD, gyrB* and *recA* gene sequences of 38 *Dickeya* strains, and *dnaX* gene sequences of 35 *Dickeya* strains were compared with sequences available in the public database (http://www.ncbi.nlm.nih.gov/). All sequences for the *atpD* (642 nucleotides), *dnaX* (536 nucleotides), *gyrB* (745 nucleotides) and *recA* (425 nucleotides) genes were edited and aligned using the ClustalW 1.6 software with parameters as: Gap Opening Penalty of 15, and Gap Extension Penalty of 6.66 for Pairwise and Multiple Alignment; Transition Weight of 0.5; and Delay Divergent Cut-off of 30%. Trees obtained with the concatenated data set of the four genes were constructed with *Pectobacterium atrosepticum* SCRI1043 as an outgroup. Method of Neighbor-joining was used for analysis, and 1,000 bootstrap replicates were included in a heuristic search, with a random tree and the tree bisection-reconnection branch-swapping algorithm. The percent variation was calculated by comparing all isolates to the nearest relative.

### Genome comparisons

According to the phylogenetic analysis, we selected the four closely relative species with released whole genomes including *D. chrysanthemi* Ech1591 (formerly named *D. zeae*, GenBank accession number CP001655.1), *D. zeae* Ech586 (formerly named *D. dadantii*, CP001836, GCF_000025065), *D. dadantii* 3937 (CP002038) and *D. paradisiaca* Ech703 (formerly named *D. dadantii*, CP001654) [10], and another two *D. zeae* rice strains including ZJU1202 (GCF_000264075) [15] and DZ2Q (GCF_000404105) [16] for genome comparisons. The sequence of EC1 is ordered according to that of the reference bacterium based on Mummer 3.22 (http://mummer.sourceforge.net/). Then the upper and following axes of linear synteny graph are constructed after the same proportion of size reduction in length of both sequences (parameter: b, 200; c, 65; extend: l, 20). According to BLAST results, each pair nucleic acid sequence of two alignments is marked in the coordinate diagram according to its position information after the same proportion of size reduction.

To identify the set of common genes and the set of genes unique to strain EC1, OrthoMCL clustering analyses were performed with the following parameters: P-value Cut-off = 1 × 10^−5^; Identity Cut-off = 90%; Percent Match Cut-off = 80.

### Ethics statement

All the experiments were conducted according to the experiment security regulations of South China Agricultural University (SCAU), and approved by the biosafety committee in SCAU.

## Availability of supporting data

The data sets supporting the results of this article are included within the article and its additional files.

## Additional files

Additional file 1:
**Phylogenetic tree based on the concatenated nucleotide sequences of the** ***atpD*** **(A),** ***dnaX*** **(B),** ***gyrB*** **(C) and** ***recA*** **(D) housekeeping genes using maximum likelihood method.** The tree was constructed with *P. atrosepticum* SCRI1043 as outgroup, and generated with 1000 bootstrap replicates. Additional file 2:
**Characteristics of the unique proteins with known function in** ***D. zeae*** **EC1 compared with other two** ***D. zeae*** **rice strains.**
Additional file 3:
**Physical map of type II secretion system in** ***Dickeya*** **spp.** Arrows denote putative transcriptional units. Open arrow indicates conserved ORF. Filled arrow indicates inserted genes. Additional file 4:
**Similarity and variations of Hrp Proteins in** ***Dickeya*** **spp.**
Additional file 5:
**Genetic organization of T4SS genes in** ***Dickeya*** **spp.**
Additional file 6:
**Physical map of flagellar associate genes and chemotaxis associate genes in** ***D. zeae*** **EC1.** Arrows denote putative transcriptional units. The dashed line on the top indicates the 9 fatty acid biosynthesis genes ranging from *W909_12275* to *W909_12315*, and the dashed line on the bottom indicates the 9 chemotaxis-associated genes ranging from *W909_12510* to *W909_12550*. Additional file 7:
**Characteristics of the fatty acid genes inserted in the** ***fli*** **gene cluster of** ***D. zeae*** **EC1.**
Additional file 8:
**Conservation of the common quorum sensing related proteins in** ***Dickeya*** **spp*** *.* *The data in brackets indicates the number of amino acids in peptide. Additional file 9:
**The Vfm homologs in** ***Dickeya*** **spp*.** *The sequences of *vfm* gene cluster are available at the asap database: http://asap.ahabs.wisc.edu/asap/ASAP1.htm. The data in brackets indicates the number of amino acids in peptide. Additional file 10:
**Vfm quorum sensing genes in** ***Dickeya*** **spp.**
Additional file 11:
**Homology analysis of the** ***zms*** **genes of** ***D. zeae*** **EC1,** ***S. plymuthica*** **AS12 and** ***D. solani*** **D s0432-1.** **zmn23* is predicted to encode a NifA subfamily transcriptional regulator, and its homolog in the genomes of *S. plymuthica* AS12 (CP002774.1), AS9 (CP002773.1) and AS13 (CP002775.1) (NCBI accession No.: AEF52351.1, AEF47399.1 and AEG30058.1), encoding a product of 684 aa, is located far from the zeamine biosynthesis gene cluster.
