# Supplementary material for: The complete genome sequence of Dickeya zeae EC1 reveals substantial divergence from other Dickeya strains and species
Source: BMC Genomics. 2015 Aug 4;16(1):571. doi: 10.1186/s12864-015-1545-x (PMC4522980; doi:10.1186/s12864-015-1545-x)
Supplement: Additional file 1: — Phylogenetic tree based on the concatenated nucleotide sequences of the atpD (A), dnaX (B), gyrB (C) and recA (D) housekeeping genes using maximum likelihood method. The tree was constructed with P. atrosepticum SCRI1043 as outgroup, and generated with 1000 bootstrap replicates. [file 12864_2015_1545_MOESM1_ESM.ppt]

## Slide 1
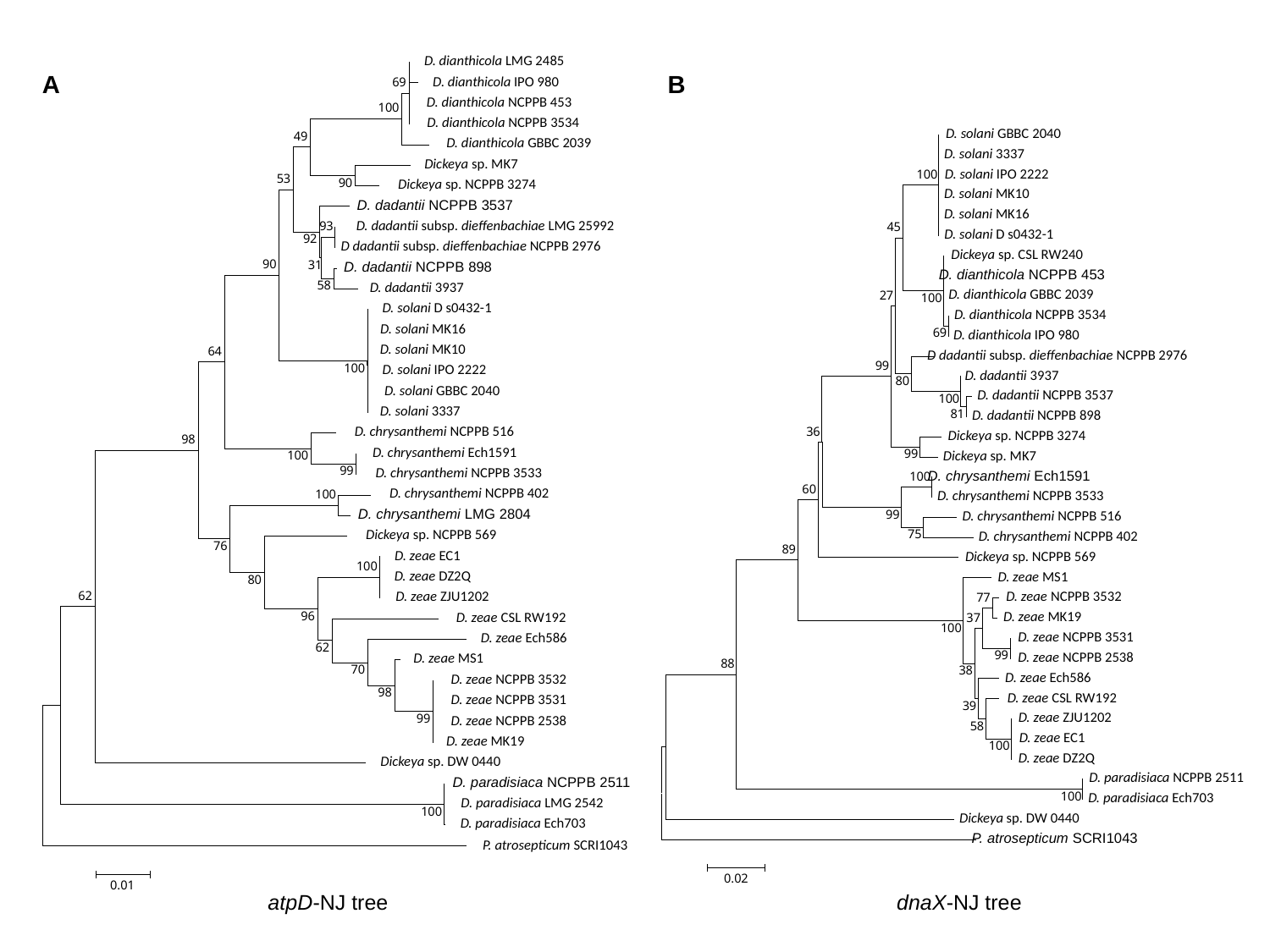

D. dianthicola LMG 2485
 D. dianthicola IPO 980
69
 D. dianthicola NCPPB 453
100
 D. dianthicola NCPPB 3534
49
 D. dianthicola GBBC 2039
 Dickeya sp. MK7
53
90
 Dickeya sp. NCPPB 3274
 D. dadantii NCPPB 3537
 D. dadantii subsp. dieffenbachiae LMG 25992
93
92
 D dadantii subsp. dieffenbachiae NCPPB 2976
90
31
 D. dadantii NCPPB 898
58
 D. dadantii 3937
 D. solani D s0432-1
 D. solani MK16
 D. solani MK10
64
100
 D. solani IPO 2222
 D. solani GBBC 2040
 D. solani 3337
 D. chrysanthemi NCPPB 516
98
 D. chrysanthemi Ech1591
100
99
 D. chrysanthemi NCPPB 3533
 D. chrysanthemi NCPPB 402
100
 D. chrysanthemi LMG 2804
 Dickeya sp. NCPPB 569
76
 D. zeae EC1
100
 D. zeae DZ2Q
80
 D. zeae ZJU1202
62
 D. zeae CSL RW192
96
 D. zeae Ech586
62
 D. zeae MS1
70
 D. zeae NCPPB 3532
98
 D. zeae NCPPB 3531
99
 D. zeae NCPPB 2538
 D. zeae MK19
 Dickeya sp. DW 0440
 D. paradisiaca NCPPB 2511
 D. paradisiaca LMG 2542
100
 D. paradisiaca Ech703
 P. atrosepticum SCRI1043
0.01
B
A
 D. solani GBBC 2040
 D. solani 3337
 D. solani IPO 2222
100
 D. solani MK10
 D. solani MK16
45
 D. solani D s0432-1
 Dickeya sp. CSL RW240
 D. dianthicola NCPPB 453
 D. dianthicola GBBC 2039
27
100
 D. dianthicola NCPPB 3534
69
 D. dianthicola IPO 980
 D dadantii subsp. dieffenbachiae NCPPB 2976
99
 D. dadantii 3937
80
 D. dadantii NCPPB 3537
100
81
 D. dadantii NCPPB 898
36
 Dickeya sp. NCPPB 3274
99
 Dickeya sp. MK7
 D. chrysanthemi Ech1591
100
60
 D. chrysanthemi NCPPB 3533
99
 D. chrysanthemi NCPPB 516
75
 D. chrysanthemi NCPPB 402
89
 Dickeya sp. NCPPB 569
 D. zeae MS1
 D. zeae NCPPB 3532
77
 D. zeae MK19
37
100
 D. zeae NCPPB 3531
99
 D. zeae NCPPB 2538
88
38
 D. zeae Ech586
 D. zeae CSL RW192
39
 D. zeae ZJU1202
58
 D. zeae EC1
100
 D. zeae DZ2Q
 D. paradisiaca NCPPB 2511
100
 D. paradisiaca Ech703
 Dickeya sp. DW 0440
 P. atrosepticum SCRI1043
0.02
atpD-NJ tree
dnaX-NJ tree

## Slide 2
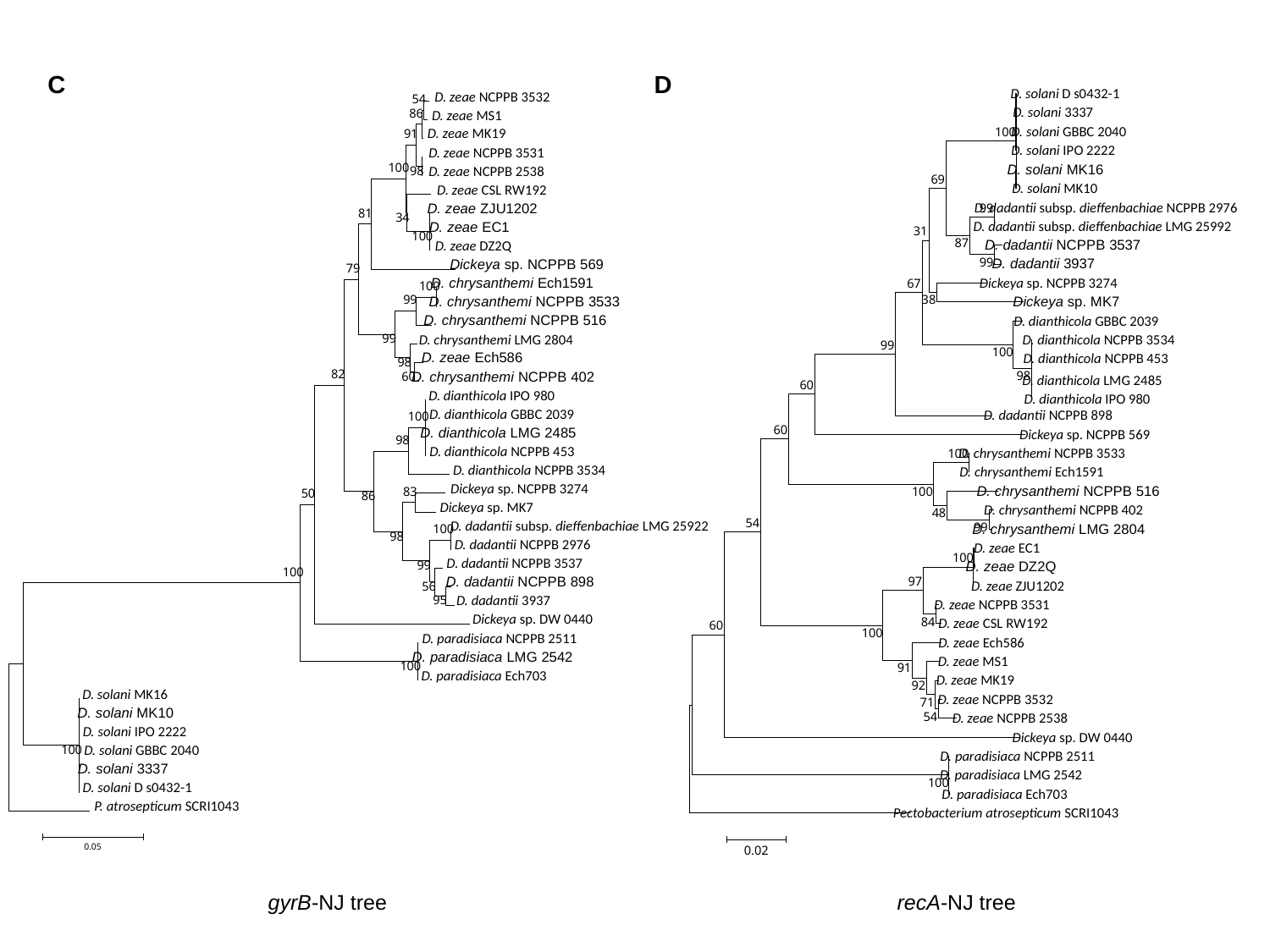

C
D
 D. solani D s0432-1
 D. solani 3337
 D. solani GBBC 2040
100
 D. solani IPO 2222
 D. solani MK16
69
 D. solani MK10
 D. dadantii subsp. dieffenbachiae NCPPB 2976
99
 D. dadantii subsp. dieffenbachiae LMG 25992
31
87
 D. dadantii NCPPB 3537
99
 D. dadantii 3937
 Dickeya sp. NCPPB 3274
67
38
 Dickeya sp. MK7
 D. dianthicola GBBC 2039
 D. dianthicola NCPPB 3534
99
100
 D. dianthicola NCPPB 453
98
 D. dianthicola LMG 2485
60
 D. dianthicola IPO 980
 D. dadantii NCPPB 898
60
 Dickeya sp. NCPPB 569
 D. chrysanthemi NCPPB 3533
100
 D. chrysanthemi Ech1591
 D. chrysanthemi NCPPB 516
100
 D. chrysanthemi NCPPB 402
48
54
99
 D. chrysanthemi LMG 2804
 D. zeae EC1
100
 D. zeae DZ2Q
97
 D. zeae ZJU1202
 D. zeae NCPPB 3531
84
 D. zeae CSL RW192
60
100
 D. zeae Ech586
 D. zeae MS1
91
 D. zeae MK19
92
 D. zeae NCPPB 3532
71
54
 D. zeae NCPPB 2538
 Dickeya sp. DW 0440
 D. paradisiaca NCPPB 2511
 D. paradisiaca LMG 2542
100
 D. paradisiaca Ech703
 Pectobacterium atrosepticum SCRI1043
0.02
 D. zeae NCPPB 3532
54
86
 D. zeae MS1
 D. zeae MK19
91
 D. zeae NCPPB 3531
100
 D. zeae NCPPB 2538
98
 D. zeae CSL RW192
 D. zeae ZJU1202
81
34
 D. zeae EC1
100
 D. zeae DZ2Q
 Dickeya sp. NCPPB 569
79
 D. chrysanthemi Ech1591
100
99
 D. chrysanthemi NCPPB 3533
 D. chrysanthemi NCPPB 516
 D. chrysanthemi LMG 2804
99
 D. zeae Ech586
98
82
 D. chrysanthemi NCPPB 402
60
 D. dianthicola IPO 980
 D. dianthicola GBBC 2039
100
 D. dianthicola LMG 2485
98
 D. dianthicola NCPPB 453
 D. dianthicola NCPPB 3534
 Dickeya sp. NCPPB 3274
83
50
86
 Dickeya sp. MK7
 D. dadantii subsp. dieffenbachiae LMG 25922
100
98
 D. dadantii NCPPB 2976
 D. dadantii NCPPB 3537
99
100
 D. dadantii NCPPB 898
56
 D. dadantii 3937
95
 Dickeya sp. DW 0440
 D. paradisiaca NCPPB 2511
 D. paradisiaca LMG 2542
100
 D. paradisiaca Ech703
 D. solani MK16
 D. solani MK10
 D. solani IPO 2222
 D. solani GBBC 2040
100
 D. solani 3337
 D. solani D s0432-1
 P. atrosepticum SCRI1043
0.05
gyrB-NJ tree
recA-NJ tree
