# Supplementary material for: The complete genome sequence of Dickeya zeae EC1 reveals substantial divergence from other Dickeya strains and species
Source: BMC Genomics. 2015 Aug 4;16(1):571. doi: 10.1186/s12864-015-1545-x (PMC4522980; doi:10.1186/s12864-015-1545-x)
Supplement: Additional file 2: — Characteristics of the unique proteins with known function in D. zeae EC1 compared with other two D. zeae rice strains. [file 12864_2015_1545_MOESM2_ESM.doc]

| **Locus tag** | **Accession no.** | **Size (aa)** | **Identity** | **Protein prediction** |
| --- | --- | --- | --- | --- |
| 457403~457603 nt | / | 66 | 98% to ACT08493.1 of Ech1591 | Insertion element protein InsA |
| W909_02140 | AJC64970.1 | 134 | 99% to ACT08488.1 of Ech1591 | Pirin |
| W909_03400 | AJC65190.1 | 63 | 82% to KFF72896.1 of *P. carotovorum* subsp. *brasiliense* CFIA1033 | Transposase |
| W909_03495 | AJC65197.1 | 73 | 96% to ACT08206.1 of Ech1591 | Transposase IS3/IS911 family protein |
| W909_07500 | AJC65916.1 | 74 | 95% to ACZ77410.1 of Ech586 | Citrate lyase acyl carrier protein CitD |
| W909_02690 | AJC65071.1 | 50 | 92% to ACT08885.1 of Ech1591 | Transposase and inactivated derivatives |
