# Supplementary figures and images for: The complete genome sequence of Dickeya zeae EC1 reveals substantial divergence from other Dickeya strains and species
Source: BMC Genomics. 2015 Aug 4;16(1):571. doi: 10.1186/s12864-015-1545-x (PMC4522980; doi:10.1186/s12864-015-1545-x)

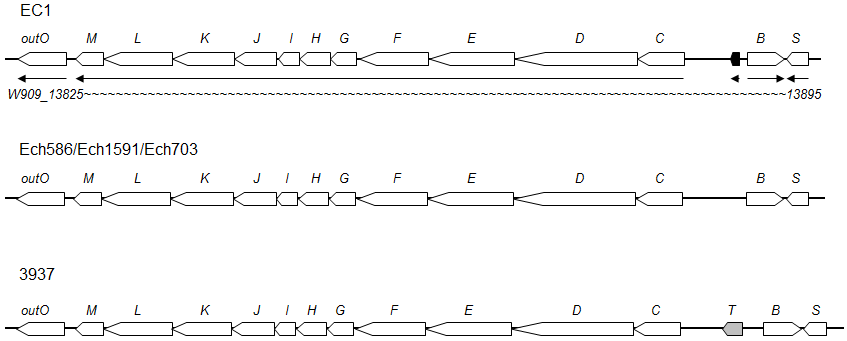

Supplement: Additional file 3: — Physical map of type II secretion system in Dickeya spp. Arrows denote putative transcriptional units. Open arrow indicates conserved ORF. Filled arrow indicates inserted genes. [file 12864_2015_1545_MOESM3_ESM.png]

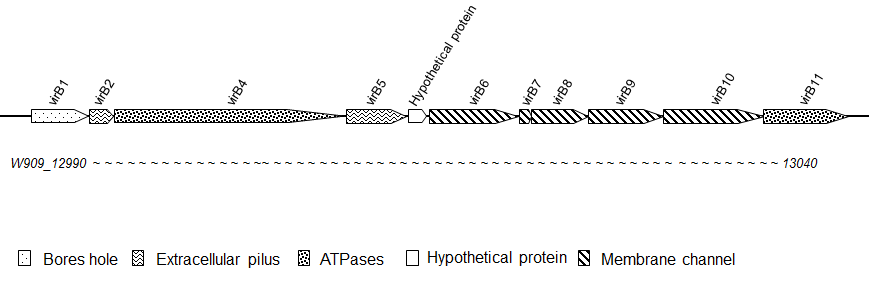

Supplement: Additional file 5: — Genetic organization of T4SS genes in Dickeya spp. [file 12864_2015_1545_MOESM5_ESM.png]

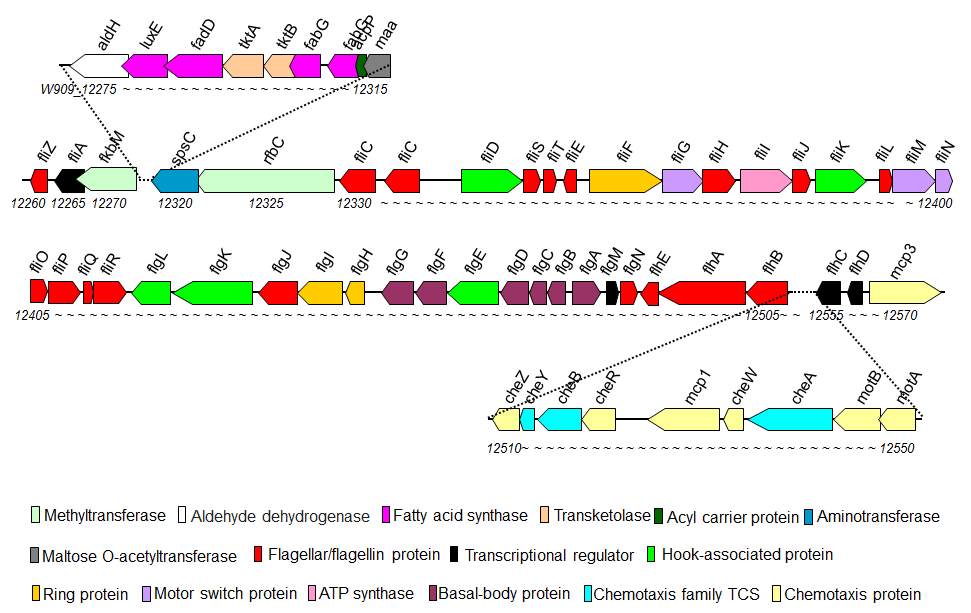

Supplement: Additional file 6: — Physical map of flagellar associate genes and chemotaxis associate genes in D. zeae EC1. Arrows denote putative transcriptional units. The dashed line on the top indicates the 9 fatty acid biosynthesis genes ranging from W909_12275 to W909_12315, and the dashed line on the bottom indicates the 9 chemotaxis-associated genes ranging from W909_12510 to W909_12550. [file 12864_2015_1545_MOESM6_ESM.png]

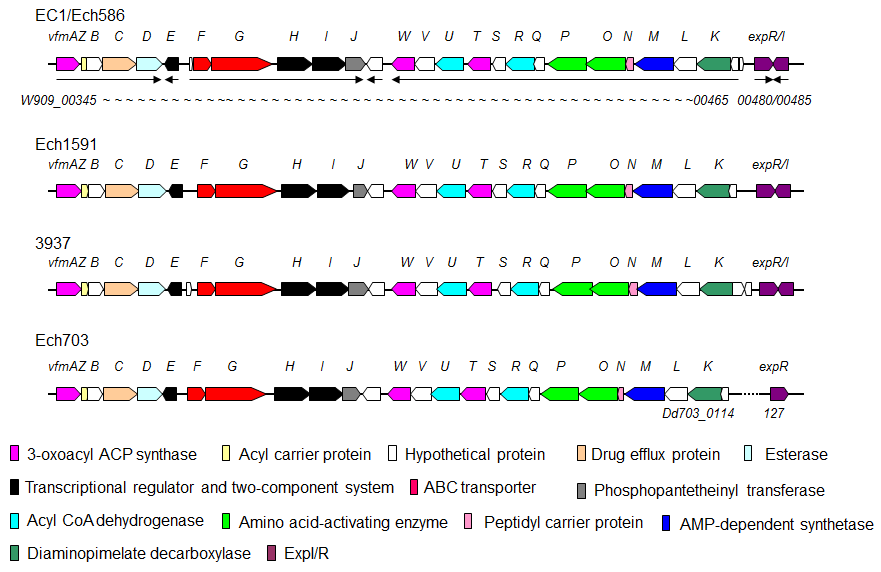

Supplement: Additional file 10: — Vfm quorum sensing genes in Dickeya spp. [file 12864_2015_1545_MOESM10_ESM.png]
