# Supplementary material for: The complete genome sequence of Dickeya zeae EC1 reveals substantial divergence from other Dickeya strains and species
Source: BMC Genomics. 2015 Aug 4;16(1):571. doi: 10.1186/s12864-015-1545-x (PMC4522980; doi:10.1186/s12864-015-1545-x)
Supplement: Additional file 4: — Similarity and variations of Hrp Proteins in Dickeya spp. [file 12864_2015_1545_MOESM4_ESM.doc]

| **Genes in EC1** | **Locus tag in EC1 (bps)** | **Ech586** | | **Ech1591** | | **3937** | | **Protein characteristics** |
| --- | --- | --- | --- | --- | --- | --- | --- | --- |
|  |  | **Locus tag (bps)** | **Homology** | **Locus tag (bps)** | **Homology** | **Locus tag (bps)** | **Homology** |  |
| ***hrpN*** | *W909_10690* (975) | *Dd586_1909* (969) | 92**%** | *Dd1591_1910* (1017) | 79**%** | *Dda3937_04152* (1029) | 77**%** | Elicitor gene, multicellular pellicle formation, cell aggregation [30,31] |
| ***hrpV*** | *W909_10695* (372) | *Dd586_1908* (402*)* | 91**%** | *Dd1591_1909* (411) | 63**%** | *Dda3937_04151* (372) | 87**%** | T3SS negative regulator |
| ***hrpT*** | *W909_10700* (192) | *Dd586_1907* (192) | 95**%** | *Dd1591_1908* (186) | 77**%** | *Dda3937_04207* (189) | 79**%** | Putative chaperone for hrcC; T3SS lipoprotein |
| ***hrcC*** | *W909_10705* (2073) | *Dd586_1906* (2073) | 97**%** | *Dd1591_1907* (2076) | 89**%** | *Dda3937_04208* (2070) | 91**%** | Outermembrane; transport [27] |
| ***hrpG*** | *W909_10710* (435) | *Dd586_1905* (435) | 99**%** | *Dd1591_1906* (435) | 86**%** | *Dda3937_04209* (435) | 90**%** | T3SS secretion gene |
| ***hrpF*** | *W909_10715* (228) | *Dd586_1904* (231) | 97**%** | *Dd1591_1905* (231) | 91**%** | *Dda3937_04210* (231) | 89**%** | T3SS secretion gene |
| ***plcA*** | *W909_10720* (1050) | *Dd586_1901* (1050) | 96**%** | *Dd1591_1904* (1050) | 83**%** | *Dda3937_04211* (1050) | 93**%** | Extracellular phospholipase |
| ***hrpE*** | *W909_10725* (609) | *Dd586_1898* (609) | 93**%** | *Dd1591_1901* (603) | 82**%** | *Dda3937_03340* (603) | 79**%** | T3SS secretion gene |
| ***hrpD*** | *W909_10730* (588) | *Dd586_1897* (583) | 83**%** | *Dd1591_1900* (564) | 67**%** | *Dda3937_03341* (651) | 68**%** | T3SS secretion gene |
| ***hrcJ*** | *W909_10735* (807) | *Dd586_1896* (807) | 97**%** | *Dd1591_1899* (786) | 93**%** | *Dda3937_03342* (711) | 94**%** | Secretion apparatus lipoprotein [30] |
| ***hrpB*** | *W909_10740* (366) | *Dd586_1895* (378) | 90**%** | *Dd1591_1898* (369) | 80**%** | *Dda3937_03343* (402) | 80**%** | Secretion apparatus lipoprotein [30] |
| ***hrpA*** | *W909_10745* (177) | *Dd586_1894* (204) | 97**%** | *Dd1591_1897* (204) | 74**%** | *Dda3937_03344* (204) | 74**%** | Structural gene [30] |
| ***hrpS*** | *W909_10750* (894) | *Dd586_1893* (978) | 97**%** | *Dd1591_1896* (978) | 92**%** | *Dda3937_03345* (978) | 90**%** | Enhancer-binding protein [28, 30] |
| ***hrpY*** | *W909_10755* (642) | *Dd586_1892* (642) | 100**%** | *Dd1591_1895* (642) | 96**%** | *Dda3937_03346* (642) | 95**%** | Two-component response regulator [30] |
| ***hrpX*** | *W909_10760* (1473) | *Dd586_1891* (1467) | 98**%** | *Dd1591_1894* (1641) | 90**%** | *Dda3937_03347* (363) | 90**%** | Two-component sensor kinase [30] |
| ***hrpL*** | *W909_10765* (555) | *Dd586_1890* (555) | 97**%** | *Dd1591_1893* (555) | 90**%** | *Dda3937_00607* (555) | 89**%** | Sigma factor [28, 30] |
| ***hrpJ*** | *W909_10770* (1155) | *Dd586_1889* (1155) | 96**%** | *Dd1591_1892* (1182) | 87**%** | *Dda3937_00608* (1170) | 87**%** | T3SS secretion regulator YopN/LcrE/InvE/MxiC |
| ***hrcV*** | *W909_10775* (2103) | *Dd586_1888* (2103) | 97**%** | *Dd1591_1891* (2103) | 94**%** | *Dda3937_00609* (2103) | 93**%** | T3SS inner membrane channel protein |
| ***hrpQ*** | *W909_10780* (984) | *Dd586_1887* (972) | 85**%** | *Dd1591_1890* (972) | 79**%** | *Dda3937_00610* (972) | 80**%** | Secretion apparatus H+-transporting two-sector ATPase |
| ***hrcN*** | *W909_10785* (1383) | *Dd586_1886* (1383) | 96**%** | *Dd1591_1889* (1380) | 92**%** | *Dda3937_00611* (1383) | 92**%** | T3SS secretion gene |
| ***hrpO*** | *W909_10790* (462) | *Dd586_1885* (480) | 92**%** | *Dd1591_1888* (480) | 86**%** | *Dda3937_00612* (480) | 84**%** | T3SS secretion gene |
| ***hrpP*** | *W909_10795* (552) | *Dd586_1884* (552) | 87**%** | *Dd1591_1887* (531) | 60**%** | *Dda3937_00613* (525) | 60**%** | T3SS secretion gene |
| ***hrcQ*** | *W909_10800* (1203) | *Dd586_1883* (1185) | 86**%** | *Dd1591_1886* (1164) | 68**%** | *Dda3937_00614* (1149) | 65**%** | T3SS secretion apparatus gene |
| ***hrcR*** | *W909_10805* (654) | *Dd586_1882* (654) | 97**%** | *Dd1591_1885* (654) | 94**%** | *Dda3937_00615* (654) | 95**%** | Inner membrane protein |
| ***hrcS*** | *W909_10810* (261) | *Dd586_1881* (261) | 99**%** | *Dd1591_1884* (261) | 97**%** | *Dda3937_00616* (261) | 95**%** | Inner membrane protein |
| ***hrcT*** | *W909_10815* (804) | *Dd586_1880* (804) | 97**%** | *Dd1591_1883* (804) | 92**%** | *Dda3937_00617* (804) | 93**%** | Component of the T3SS [28] |
| ***hrcU*** | *W909_10820* 1080) | *Dd586_1879* (1080) | 97**%** | *Dd1591_1882* (1186) | 88**%** | *Dda3937_00618* (1180) | 89**%** | Core component of theT3SS |
