# Supplementary material for: The complete genome sequence of Dickeya zeae EC1 reveals substantial divergence from other Dickeya strains and species
Source: BMC Genomics. 2015 Aug 4;16(1):571. doi: 10.1186/s12864-015-1545-x (PMC4522980; doi:10.1186/s12864-015-1545-x)
Supplement: Additional file 7: — Characteristics of the fatty acid genes inserted in the fli gene cluster of D. zeae EC1. [file 12864_2015_1545_MOESM7_ESM.doc]

| **Gene** | **Accession no. (aa)** | **Identity** | **Protein characteristics** |
| --- | --- | --- | --- |
| *aldH* | AJC66806.1 (404) | 49% to BAK11662.1 in *Pantoea ananatis* AJ13355 | Acyl-CoA reductase |
| *luxE* | AJC66807.1 (359) | 59% to BAK11663.1 | Long-chain-fatty-acid--luciferin-component ligase |
| *fadD* | AJC66808.1 (468) | 47% to BAK11664.1 | Long-chain-fatty-acid--CoA ligase FadD |
| *tktA* | AJC66809.1 (322) | 70% to BAK11665.1 | Transketolase, C-terminal subunit |
| *tktB* | AJC66810.1 (271) | 63% to BAK11666.1 | Transketolase, N-terminal subunit |
| *fabG* | AJC66811.1 (248) | 69% to BAK11667.1 | 3-oxoacyl-ACP reductase FabG |
| *fabG* | AJC66812.1 (247) | 66% to BAK11668.1 | 3-oxoacyl-ACP reductase FabG |
| *acpP* | AJC66813.1 (78) | 74% to ADD77443.1 in *P. ananatis* LMG 20103 | AcpP; Acyl carrier protein |
| *maa* | AJC66814.1 (218) | 55% to BAK11670.1 | Maltose O-acetyltransferase |
