# Supplementary material for: The complete genome sequence of Dickeya zeae EC1 reveals substantial divergence from other Dickeya strains and species
Source: BMC Genomics. 2015 Aug 4;16(1):571. doi: 10.1186/s12864-015-1545-x (PMC4522980; doi:10.1186/s12864-015-1545-x)
Supplement: Additional file 8: — Conservation of the common quorum sensing related proteins in Dickeya spp* . *The data in brackets indicates the number of amino acids in peptide. [file 12864_2015_1545_MOESM8_ESM.doc]

| **Protein** | **Locus tag in EC1** | **Ech586** | | **Ech1591** | | **3937** | | **Ech703** | | **Protein characteristics** |
| --- | --- | --- | --- | --- | --- | --- | --- | --- | --- | --- |
|  |  | **Locus tag** | **Homology** | **Locus tag** | **Homology** | **Locus tag** | **Homology** | **Locus tag** | **Homology** |  |
| ExpR | W909_00480 (250) | Dd586_0111  (250) | 97% | Dd1591_0100  (250) | 91% | Dda3937_03218  (250) | 91% | Dd703_0127  (236) | 64% | LuxR family transcriptional regulator ExpR |
| ExpI | W909_00485 (212) | Dd586_0112  (212) | 99% | Dd1591_0101  (212) | 95% | Dda3937_03219  (212) | 93% | / | / | Acyl-homoserine-lactone synthase ExpI |
| LuxS | W909_4510 (158) | Dd586_3179  (171) | 99% | Dd1591_0988  (171) | 96% | Dda3937_03941  (171) | 97% | Dd703_1003  (171) | 89% | LuxS S-ribosylhomocysteine lyase |
