# Supplementary material for: The complete genome sequence of Dickeya zeae EC1 reveals substantial divergence from other Dickeya strains and species
Source: BMC Genomics. 2015 Aug 4;16(1):571. doi: 10.1186/s12864-015-1545-x (PMC4522980; doi:10.1186/s12864-015-1545-x)
Supplement: Additional file 9: — The Vfm homologs in Dickeya spp*. *The sequences of vfm gene cluster are available at the asap database: http://asap.ahabs.wisc.edu/asap/ASAP1.htm. The data in brackets indicates the number of amino acids in peptide. [file 12864_2015_1545_MOESM9_ESM.doc]

| **3937** | | | **EC1** | | **Ech586** | | **Ech1591** | | **Ech703** | |
| --- | --- | --- | --- | --- | --- | --- | --- | --- | --- | --- |
| **Protein** | **Locus tag** | **Putative function** | **Locus tag** | **Identity** | **Locus tag** | **Identity** | **Locus tag** | **Identity** | **Locus tag** | **Identity** |
| **VfmA** | Dda3937_00971(318) | 3-oxoacyl-ACP synthase | W909_00345 (318) | 88% | Dd586_0085(323) | 87% | Dd1591_0074(334) | 90% | Dd703_0089(325) | 73% |
| **VfmZ** | Dda3937_00970(88) | Acyl carrier protein | W909_00350 (88) | 92% | Dd586_0086(88) | 92% | Dd1591_0075(88) | 90% | Dd703_0090(88) | 78% |
| **VfmB** | Dda3937_00969(203) |  | W909_00355 (203) | 85% | Dd586_0087(203) | 85% | Dd1591_0076(217) | 81% | Dd703_0091(203) | 76% |
| **VfmC** | Dda3937_00968(451) | Drug efflux protein | W909_00360 (451) | 87% | Dd586_0088(451) | 87% | Dd1591_0077(452) | 89% | Dd703_0092(453) | 71% |
| **VfmD** | Dda3937_0096(362) | Esterase | W909_00365 (343) | 83% | Dd586_0089(343) | 86% | Dd1591_0078(363) | 84% | Dd703_0093(333) | 71% |
| **VfmE** | Dda3937_00966(187) | Transcriptional regulator | W909_00370 (187) | 94% | Dd586_0090(187) | 94% | Dd1591_0079(187) | 95% | Dd703_0094(185) | 86% |
| **VfmF** | Dda3937_00965(235) | ABC transporter ATPase | W909_00375 (235) | 98% | Dd586_0091(235) | 98% | Dd1591_0080(235) | 97% | Dd703_0095(235) | 94% |
| **VfmG** | Dda3937_00964(811) | ABC transporter permease | W909_00380 (811) | 83% | Dd586_0092(813) | 61% | Dd1591_0081(810) | 83% | Dd703_0096(813) | 58% |
| **VfmH** | Dda3937_00963(466) | Two component RR | W909_00385 (466) | 95% | Dd586_0093(466) | 95% | Dd1591_0082(466) | 95% | Dd703_0097(466) | 88% |
| **VfmI** | Dda3937_00962(439) | Two component sensor | W909_00390 (439) | 94% | Dd586_0094(439) | 96% | Dd1591_0083(426) | 94% | Dd703_0098(439) | 87% |
| **VfmJ** | Dda3937_00961(256) | 4'-phosphopantetheinyl transferase | W909_00395 (266) | 70% | Dd586_0095(257) | 74% | Dd1591_0084(195) | 80% | Dd703_0099(248) | 53% |
| **VfmK** | Dda3937_03216(440) | Orn/DAP/Arg decarboxylase 2 | W909_00465 (446) | 85% | Dd586_0109(446) | 83% | Dd1591_0098(440) | 83% | Dd703_0113(442) | 74% |
| **VfmL** | Dda3937_03215(303) |  | W909_00460 (309) | 73% | Dd586_0108(309) | 75% | Dd1591_0097(306) | 78% | Dd703_0112(305) | 65% |
| **VfmM** | Dda3937_03214(527) | AMP-dependent synthetase | W909_00455 (527) | 88% | Dd586_0107(527) | 89% | Dd1591_0096(527) | 88% | Dd703_0111(526) | 79% |
| **VfmN** | Dda3937_03213(102) | Peptidyl carrier protein | W909_00450 (102) | 81% | Dd586_0106(102) | 81% | Dd1591_0095(102) | 84% | Dd703_0110(90) | 67% |
| **VfmO** | Dda3937_03212(514) | Amino acid-activating enzyme | W909_00445 (522) | 78% | Dd586_0105(522) | 78% | Dd1591_0094(519) | 81% | Dd703_0109(516) | 64% |
| **VfmP** | Dda3937_03211(532) | Amino acid-activating enzyme | W909_00440 (517) | 81% | Dd586_0104(515) | 63% | Dd1591_0093(510) | 80% | Dd703_0108(506) | 55% |
| **VfmQ** | Dda3937_03210(143) |  | W909_00435 (143) | 93% | Dd586_0103(143) | 93% | Dd1591_0092(143) | 93% | Dd703_0107(143) | 83% |
| **VfmR** | Dda3937_03209(377) | Acyl-CoA dehydrogenase | W909_00430 (377) | 90% | Dd586_0102(377) | 90% | Dd1591_0091(377) | 93% | Dd703_0106(376) | 79% |
| **VfmS** | Dda3937_03208(170) |  | W909_00425 (170) | 78% | Dd586_0101(177) | 78% | Dd1591_0090(176) | 77% | Dd703_0105(172) | 54% |
| **VfmT** | Dda3937_04302(307) | 3-oxoacyl ACP synthases | W909_00420 (307) | 88% | Dd586_0100(307) | 87% | Dd1591_0089(307) | 89% | Dd703_0104(325) | 78% |
| **VfmU** | Dda3937_00957(378) | Acyl CoA dehydrogenase | W909_00415 (378) | 89% | Dd586_0099(378) | 88% | Dd1591_0088(378) | 90% | Dd703_0103(378) | 79% |
| **VfmV** | Dda3937_00958(285) |  | W909_00410 (265) | 85% | Dd586_0098(285) | 86% | Dd1591_0087(285) | 85% | Dd703_0102(278) | 58% |
| **VfmW** | Dda3937_00959(312) | 3-oxoacyl ACP synthases | W909_00405 (312) | 82% | Dd586_0097(316) | 76% | Dd1591_0086(316) | 76% | Dd703_0101(307) | 66% |
|  | Dda3937_00960(213) |  | W909_00400 (220) | 79% | Dd586_0096(220) | 80% | Dd1591_0085(215) | 80% | Dd703_0100(246) | 56% |
