# Supplementary material for: The complete genome sequence of Dickeya zeae EC1 reveals substantial divergence from other Dickeya strains and species
Source: BMC Genomics. 2015 Aug 4;16(1):571. doi: 10.1186/s12864-015-1545-x (PMC4522980; doi:10.1186/s12864-015-1545-x)
Supplement: Additional file 11: — Homology analysis of the zms genes of D. zeae EC1, S. plymuthica AS12 and D. solani D s0432-1. *zmn23 is predicted to encode a NifA subfamily transcriptional regulator, and its homolog in the genomes of S. plymuthica AS12 (CP002774.1), AS9 (CP002773.1) and AS13 (CP002775.1) (NCBI accession No.: AEF52351.1, AEF47399.1 and AEG30058.1), encoding a product of 684 aa, is located far from the zeamine biosynthesis gene cluster. [file 12864_2015_1545_MOESM11_ESM.doc]

| ***D. zeae* EC1** | | ***S. plymuthica* AS12** | | | ***D. solani* D s0432-1** | | **Proposed function** |
| --- | --- | --- | --- | --- | --- | --- | --- |
| **Protein (aa)** | **Accession No.** | **Protein (aa)** | **Accession No.** | **Homology** | **Accession No.** | **Homology** |
| N/A | N/A | Zmn1 (141) | AEF52337.1 | N/A | N/A | N/A | Hypothetical protein |
| N/A | N/A | Zmn2 (170) | AEF52336.1 | N/A | N/A | N/A | Hypothetical protein |
| N/A | N/A | Zmn3 (457) | AEF52335.1 | N/A | N/A | N/A | Pyridpxal-dependent decarboxylase |
| N/A | N/A | Zmn4 (217) | AEF52334.1 | N/A | N/A | N/A | Hypothetical protein |
| ZmsO (239) | ([AJC65774.1](http://www.ncbi.nlm.nih.gov/protein/744785522)) | Zmn5 (242) | AEF52333.1 | 62.55% | ERO58214.1 (237) | 75% | 4’-phosphopanteinyl transferase |
| ZmsP (305) | ([AJC65775.1](http://www.ncbi.nlm.nih.gov/protein/744785522)) | Zmn6 (306) | AEF52332.1 | 83.17% | ERO58215.1 (309) | 90% | HlyD family secretion protein |
| ZmsQ (239) | ([AJC65776.1](http://www.ncbi.nlm.nih.gov/protein/744785519)) | Zmn7 (239) | AEF52331.1 | 88.28% | ERO58216.1 (239) | 94% | ABC transporter ATP-binding protein |
| ZmsR (375) | ([AJC65777.1](http://www.ncbi.nlm.nih.gov/protein/744785522)) | Zmn8 (388) | AEF52330.1 | 92.97% | ERO58217.1 (375) | 91% | ABC transporter membrane protein |
| ZmsS (329) | ([AJC68376.1](http://www.ncbi.nlm.nih.gov/protein/744788119)) | Zmn9 (331) | AEF52329.1 | 65.88% | ERO58218.1 (327) | 59% | Hypothetical protein |
| ZmsA (2346) | ([AJC65778.1](http://www.ncbi.nlm.nih.gov/protein/744785522)) | Zmn10 (2259) | AEF52328.1 | 80.67% | ERO58219.1 (2312) | 74% | Polyketide synthase; PKS |
| ZmsB (1457) | ([AJC65779.1](http://www.ncbi.nlm.nih.gov/protein/744785522)) | Zmn11 (1439) | AEF52327.1 | 83.75% | ERO58220.1 (1437) | 83% | Polyunsaturated fatty acid synthase/Polyketide synthase |
| ZmsC (1003) | ([AJC65780.1](http://www.ncbi.nlm.nih.gov/protein/744785523)) | Zmn12 (1010) | AEF52326.1 | 90.99% | ERO58220.1 (1003) | 89% | Polyunsaturated fatty acid synthase, PfaD family protein |
| ZmsD (255) | ([AJC65781.1](http://www.ncbi.nlm.nih.gov/protein/744785523)) | Zmn13 (255) | AEF52325.1 | 78.55% | ERO58222.1 (255) | 91% | 3-oxoacyl-ACP reductase |
| ZmsE (411) | ([AJC65782.1](http://www.ncbi.nlm.nih.gov/protein/744785525)) | Zmn14 (412) | AEF52324.1 | 88.81% | ERO58223.1 (421) | 85% | Thioester reductase |
| ZmsF (258) | ([AJC65783.1](http://www.ncbi.nlm.nih.gov/protein/744785526)) | Zmn15 (259) | AEF52323.1 | 84.94% | ERO58224.1 (257) | 91% | Hydrolase family protein; carbon-nitrogen hydrolase |
| ZmsG (4164) | ([AJC65784.1](http://www.ncbi.nlm.nih.gov/protein/744785527)) | Zmn16 (4169) | AEF52322.1 | 50.49% | ERO58225.1 (4166) | 77% | Non-ribosomal peptides synthetase; NRPS |
| ZmsI (2163) | ([AJC65786.1](http://www.ncbi.nlm.nih.gov/protein/744785529)) | Zmn17 (2180) | AEF52321.1 | 82.47% | ERO58226.1 (2169) | 83% | Non-ribosomal peptides synthetase; NRPS |
| ZmsJ (1517) | (AJC65785.1) | Zmn18 (1531) | AEF52320.1 | 80.46% | ERO58227.1 (1517) | 79% | Polyketide synthase; PKS |
| ZmsK (497) | ([AJC65787.1](http://www.ncbi.nlm.nih.gov/protein/744785530)) | Zmn19 (439) | AEF52319.1 | 75.34% | ERO58228.1 (503) | 77% | Condensation domain-containing protein;NRPS |
| ZmsL (314) | ([AJC65788.1](http://www.ncbi.nlm.nih.gov/protein/744785532)) | Zmn20 (314) | AEF52318.1 | 92.99% | ERO58229.1 (314) | 92% | ABC transporter ATP-binding protein |
| ZmsM (370) | ([AJC65789.1](http://www.ncbi.nlm.nih.gov/protein/744785531)) | Zmn21 (371) | AEF52317.1 | 89.52% | ERO58230.1 (370) | 86% | ABC transporter permease |
| ZmsN (348) | ([AJC65790.1](http://www.ncbi.nlm.nih.gov/protein/744785533)) | Zmn22 (345) | AEF52316.1 | 81.84% | ERO58231.1 (343) | 80% | Hydrolase family protein |
| N/A | N/A | Zmn23* (106) | AEF52351.1 | N/A | ERO59136.1 (466) | 54% | NifA subfamily transcriptional regulator |
